# Synthesis of 3-Amino-4-substituted Monocyclic ß-Lactams—Important Structural Motifs in Medicinal Chemistry

**DOI:** 10.3390/ijms23010360

**Published:** 2021-12-29

**Authors:** Katarina Grabrijan, Nika Strašek, Stanislav Gobec

**Affiliations:** Faculty of Pharmacy, University of Ljubljana, Aškerčeva 7, 1000 Ljubljana, Slovenia; katarina.grabrijan@ffa.uni-lj.si (K.G.); nika.strasek@ffa.uni-lj.si (N.S.)

**Keywords:** monocyclic ß-lactam, Staudinger [2+2] cyclocondensation, cerium ammonium nitrate, hydrazine hydrate, 3-amino-4-substituted azetidin-2-one

## Abstract

Monocyclic ß-lactams (azetidin-2-ones) exhibit a wide range of biological activities, the most important of which are antibacterial, anticancer, and cholesterol absorption inhibitory activities. The synthesis of decorated monocyclic ß-lactams is challenging because their ring is highly constrained and consequently reactive, which is also an important determinant of their biological activity. We present the optimized synthesis of orthogonally protected 3-amino-4-substituted monocyclic ß-lactams. Among several possible synthetic approaches, Staudinger cycloaddition proved to be the most promising method for initial ring formation, yielding monocyclic ß-lactams with different substituents at the C-4 position, a phthalimido-protected 3-amino group, and a (dimethoxy)benzyl protected ring nitrogen. Challenging deprotection methods were then investigated. Oxidative cleavage with cerium ammonium nitrate and ammonia-free Birch reduction was found to be most effective for selective removal of ring nitrogen protection. Hydrazine hydrate was used for deprotection of the phthalimido group, and the procedure had to be modified by the addition of HCl in the case of aromatic substituents at the C-4 position. The presented methods and the synthesized 3-amino-4-substituted monocyclic ß-lactam derivatives are an important step toward new ß-lactams with potential pharmacological activities.

## 1. Introduction

Ever since Alexander Fleming’s serendipitous discovery of the first broad-spectrum ß-lactam antibiotic (i.e., penicillin G) in the late 1920s, ß-lactams arguably remain the single most clinically useful class of antibiotics discovered to date, in some countries making up over 60% of all antibiotic sales [1,2]. Their excellent safety and efficacy profiles and the highly reactive nature of the CO-N bond in the ß-lactam ring have propelled this structural motif in many drug discovery initiatives, besides their primary use as antibacterial agents [3,4,5]. Not only presence of the bicyclic ring system of penicillins is essential for their antibacterial activity, but it can also be replaced by a monocyclic ß-lactam. Appropriately decorated 3-aminoazetidin-2-ones serve as mimics of the D-Ala-D-Ala subunit of the stem peptide in the nascent peptidoglycan. ß-Lactams act as mechanism-based inhibitors of the transpeptidase activity of penicillin-binding proteins (PBPs), thus inhibiting the cross-linking step in peptidoglycan chains, ultimately leading to bacterial cell death [6,7]. Aztreonam, the first clinically approved synthetic monobactam (i.e., *N*-sulfonated monocyclic ß-lactam) in the 1980s, is still in use worldwide because of its suitable activity against Gram-negative bacteria and ß-lactamase stability [8]. Another approved monocyclic ß-lactam drug in clinical use is ezetimibe, which acts as a cholesterol absorption inhibitor and is used to treat hypercholesterolemia [9,10].

Azetidin-2-one, a fundamental building block of all ß-lactam antibiotics, is a four-membered cyclic lactam (i.e., ß-lactam ring) with an oxo group at the C-2 position and various substituents at the N-1, C-3, and C-4 positions [11]. The highly strained nature of the core ß-lactam skeleton is largely responsible for its chemical reactivity, which can be enhanced further by the nature of the substituents present on the ß-lactam ring, and in particular on the endocyclic nitrogen (N-1) [12]. Structural modifications of this scaffold with different substituents on the monocyclic ß-lactam core can lead to a wide range of biological activities [13]. Derivatives of monocyclic ß-lactams have been explored as agents for the treatment of atherosclerotic coronary heart disease, allergic and inflammatory conditions, autoimmune diseases, neurodegenerative diseases, diabetes, arterial thrombosis, microbial infections, and cancer [3,5]. Appropriately decorated azetidine-2-ones act as inhibitors of different nucleophilic enzymes, most commonly serine, cysteine, and threonine proteases [13,14]. Monocyclic ß-lactams are explored as cholesterol absorption inhibitors, activators and inhibitors of lecithin-cholesterol acyltransferase, vasopressin V1a antagonists, tryptase and chymase inhibitors, thrombin inhibitors, factor XIa or kallikrein inhibitors, cathepsin K inhibitors, 20S proteasome inhibitors, human leukocyte elastase inhibitors, beta lactam combretastatin mimetics, carbonic anhydrase inhibitors, *N*-acyl ethanolamine acid amidase inhibitors, inhibitors of dengue and West Nile virus NS2B-NS3 protease, human cytomegalovirus protease inhibitors, RORγt (retinoid-related orphan receptor gamma t) modulators and glutamate uptake modulators [3,4,5,7,15]. In addition to their own therapeutic potential, they are useful as synthetic synthons in the preparation of various other compounds in modern organic chemistry [16,17].

**Figure 1:**
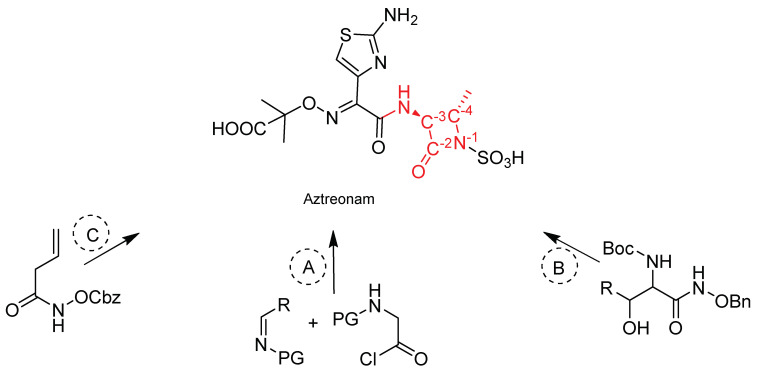
Typical synthetic routes to known C-4-substituted 3-amino-ß-lactams with antibacterial activity ((**A**) Staudinger [2+2] cyclocoaddition, (**B**) Mitsunobu-mediated cyclization, (**C**) bromine-induced cyclization).

Since the discovery of the first monocyclic ß-lactams, which were originally isolated from bacteria and were, as such, not suitable for further chemical modifications, many efforts have been directed toward the development of new synthetic methods for their production, which would enable the desirable structural diversification [7,18]. These can be broadly categorized into cycloadditions, cyclizations, and other transformations (Figure 1) [19]. Monocyclic β-lactams are most often synthesized by the Staudinger [2+2] cycloaddition between ketenes, generated in situ by treating acyl chlorides with a mild base and imines (Figure 1, reaction A) [20]. This three-step reaction cascade consists of (i) nucleophilic addition of the imine nitrogen to the electrophilic carbon of the ketene, (ii) the formation of a zwitterionic intermediate, and (iii) ring closure [21]. More recently, enolate-imine cycloaddition [22] and the Kingunsa reaction with rearrangement [23] have been studied as means to access decorated monocyclic β-lactams. Various other cyclizations involving N1-C2, C3-C4, and N1-C4 ring closures have also been reported [19,24]. In addition, there are also other less commonly used procedures, such as CO insertion [3+1] on aziridine ring, Ugi four-component reactions, and diiodomethane additions to amide dianions [25,26]. The most widely used protocols are based on either Mitsunobu-mediated cyclization of α-hydroxy-ß-amino acid hydroxamates (Figure 1, reaction B), or bromine-induced cyclization of γ,δ-unsaturated hydroxamates (Figure 1, reaction C). While both synthetic strategies rely on the acidity of the N-H in hydroxamates to facilitate the desirable cyclization and allow for a wide range of different substitutions at C-3 and C-4 of the newly formed ring, the number and complexity of synthetic steps required are often preventing synthetic success [27,28].

Further structural modifications of monocyclic ß-lactams are necessary due to emerging bacterial resistance and increased production of ß-lactamases [29], hydrolytic enzymes that can inactivate ß-lactams by hydrolysis [30]. We, therefore, believe that there is scope for improving the pharmacological profiles of azetidine-2-ones, especially in antibacterial and anticancer applications, with the introduction of appropriate substituents on the C-4 position. Herein we report our synthetic efforts to prepare a set of novel monocyclic ß-lactams using Staudinger cycloaddition reaction and studies on the optimization of the challenging N-1/N-3 deprotection reactions. The main objective of this work was, therefore, to establish a more convenient and reliable method for the synthesis of 3-amino-4-substituted azetidin-2-ones, which are important intermediates in the development of pharmacologically relevant monocyclic ß-lactams.

## 2. Results and Discussion

Our initial efforts to prepare the desired 3-amino-4-substituted monocyclic ß-lactams through N1–C4 ring closure reactions (e.g., via Mitsunobu cyclization [31] or bromine-induced cyclization [32]) were unproductive. In a subsequent approach, the convergent methodology for the stereoselective synthesis of functionalized β-lactams with a broad substrate scope developed by Staudinger et al. [20] was explored. The preparation of the ketene and imine building blocks required for the Staudinger [2+2] cycloaddition to provide target ß-lactams, as well as our studies on the necessary N-1 and N-3 deprotections, are discussed in more detail below.

### 2.1. Methods for Cyclization of 2-Azetidinone (Monocyclic Beta Lactam Core)

**Scheme 1:**
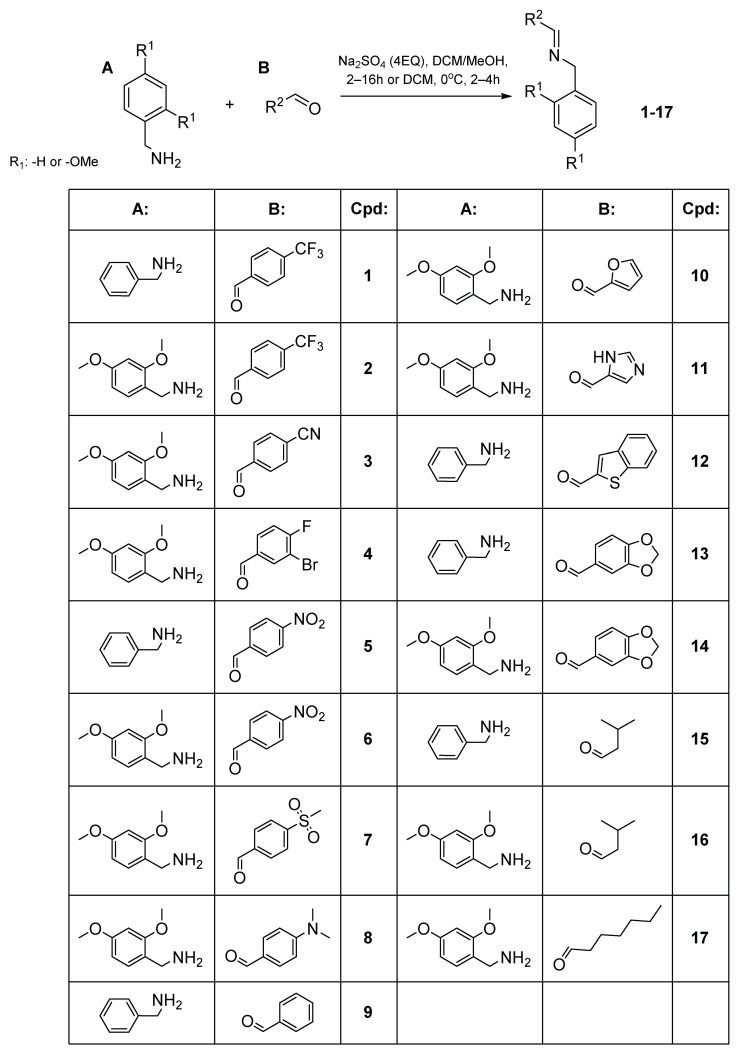
Reaction conditions for synthesis of imines and structures of products (**1–17**). Reagents and reaction conditions: Na_2_SO_4_ (4EQ), DCM/MeOH, 2–16 h or DCM, 0 °C, 2–4 h.

The requisite imines (**1–14**) were formed by the condensation of appropriate primary amines and aldehydes in dichloromethane or methanol at room temperature, using anhydrous sodium sulfate as a drying agent (Scheme 1). To demonstrate the possibility of incorporating a variety of different substituents at the C-4 position of the monocyclic ß-lactam, we selected several aldehydes from our in-house library of chemicals. The selected amines were previously described in the synthesis of monocyclic ß-lactams. In the case of the more reactive aliphatic aldehydes (**15–17**), the condensation reactions were carried out on ice, and the imines were used directly without evaporation of the solvent.

**Scheme 2:**
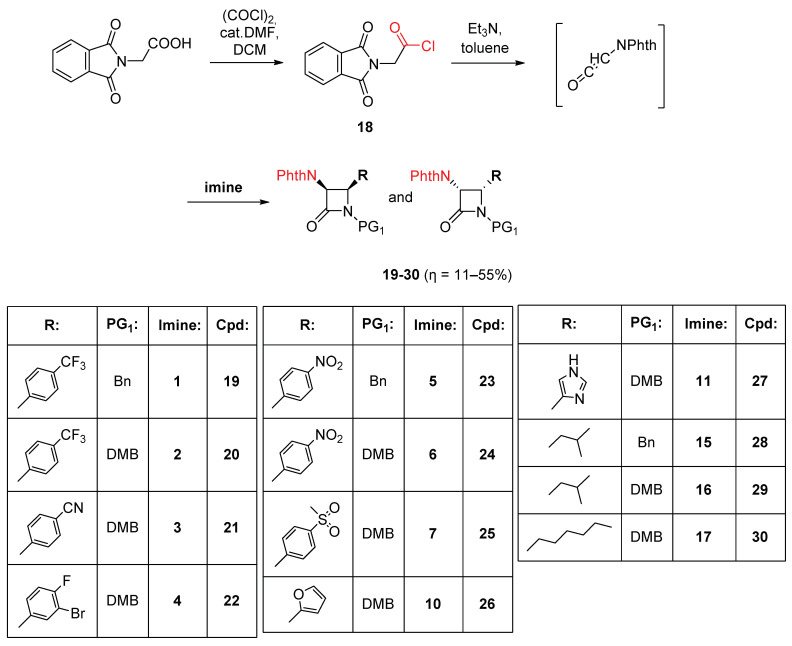
General reaction scheme for Staudinger [2+2] cycloaddition, starting from ketene, generated from acyl chloride, and a Schiff base. Bn: benzyl, DMB: 2,4-dimethoxybenzyl. Reagents and reaction conditions: 1. (COCl)_2_, cat.DMF, DCM; 2. Et_3_N, toluene.

We initially focused our efforts on the preparation of monocyclic ß-lactams using ketenes obtained from *t*-butylcarbamate- or benzylcarbamate-protected glycine and imines derived from aromatic aldehydes to test reactivity in Staudinger model reactions. However, the expected [2+2] cycloaddition products (i.e., 2-azetidinones) were not observed with any of the evaluated carbamates. This may be due to the competing formation of 1,3-oxazin-4-ones, which are highly stable and cannot react further to form 2-azetidinones [33,34]. Therefore, we have elected to use a phthalimido group to protect the glycine-amino group instead. Ketenes, prepared in situ from an acyl chloride with *N*-phthalimido protecting group (e.g., **18**), were prone to undergo the desired cycloadditions (Scheme 2). The reactions proceeded smoothly when the nitrogen of the amino acid residue was protected by substitution of both hydrogen atoms, as in the case of phthalimido-protected glycine. An acyl chloride was added dropwise to a mixture of imine and a base in toluene at 80 °C, and the product formed was easily isolated by precipitation or column chromatography. Because of the instability of ketenes, the order of the addition of the reactants was also an important factor.

The imines (**1**–**17**) were mainly obtained by the reactions of aromatic aldehydes (which were substituted by electron-withdrawing groups) with dimethoxybenzylamine or benzylamine. The products of the Staudinger reaction (**19**–**27**) in the case of an aromatic or heterocyclic substituent at the C-4 position of the ring were mainly isolated as *cis*-isomers; *trans*-stereoisomers were either not detected or were only present in traces that we could not isolate. Staudinger cycloaddition is a stepwise reaction initiated by the nucleophilic attack of an imine on a ketene, leading to a zwitterionic intermediate, followed by ring closure of this intermediate. Direct ring closure leads to the *cis*-stereoisomer, while indirect ring closure with further isomerization leads to the *trans*-stereoisomer. As previously reported in the literature, we found that electron-withdrawing groups on the imine facilitate the progress of the reaction, and electron-donating groups slow down the cyclization. Improved yields and exclusive formation of *cis*-stereoisomer were obtained with imines bearing aromatic substituents on the imine moiety, compared to imines formed from aliphatic aldehydes, which provided much lower yields and lower diastereoselectivity. The *cis-*configuration of newly synthesized monocyclic β-lactams was deduced using ^1^H NMR coupling constants (*J* values) of the β-lactam ring hydrogens H-3 and H-4; for *cis*-β-lactams *J*_3,4_ ~ 5 Hz, and for *trans-*β-lactams *J*_3,4_ ~ 2 Hz [21].

**Scheme 3:**
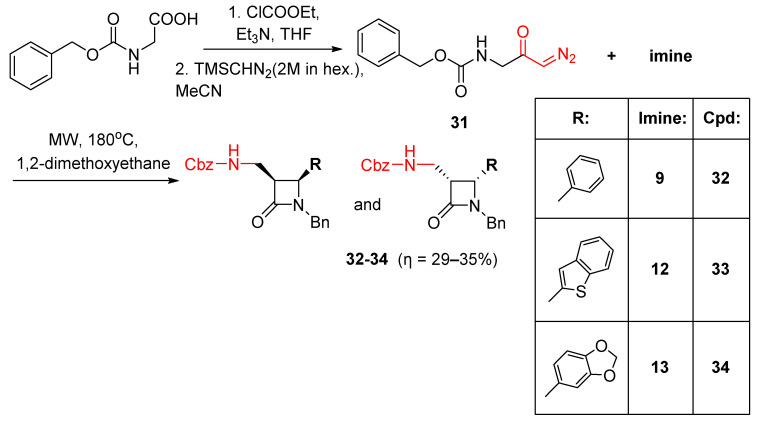
General reaction scheme for Staudinger [2+2] cyclocondensation starting from ketene, generated from diazoketone, and Schiff base under microwave radiation. Reagents and reaction conditions: 1. ClCOOEt, Et_3_N, THF; 2. TMSCHN_2_ (2 M in hex.), MeCN; 3. MW, 180 °C, 1,2-dimethoxyethane.

Since the removal of the phthalimide protecting group requires relatively harsh conditions, we opted to prepare ß-lactam analogs bearing carbamate protecting groups at the N-3 position instead, which we hoped would be more easily removed. Alternatively, functionalized 2-azetidinones can also be prepared via microwave-assisted coupling of imines with diazoketones, which can be derived from *t*-butylcarbamate- or benzylcarbamate-protected α-amino acids [33]. Such monocyclic ß-lactams are structurally different from analogous derivatives prepared via the previously described acyl chloride method by having an additional methylene unit present at C-3 of the ß-lactam ring (Scheme 3). The monocyclic ß-lactams (**32**–**34**), which were prepared using this methodology, were isolated as *trans-*isomers, as opposed to the otherwise *cis-*isomers, which are formed via, e.g., Staudindger synthesis. A significant disadvantage of this method is the preparation of diazoketone (**31**), as most methods require the use of highly toxic diazomethane or expensive trimethylsilyldiazometane [35].

**Scheme 4:**
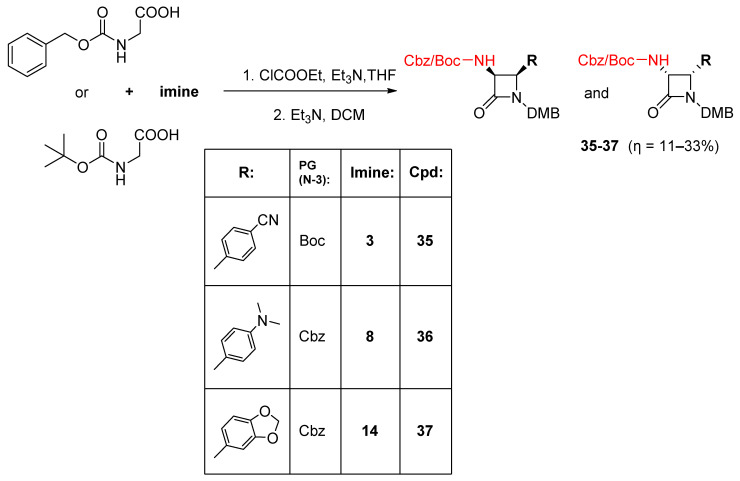
General reaction scheme for Staudinger [2+2] cyclocondensation starting from ketene, generated from mixed anhydride, and Schiff base. Reagents and reaction conditions: 1. ClCOOEt, Et_3_N, THF; 2. Et_3_N, DCM.

Finally, another convenient method was used to synthesize the ß-lactam ring from *t*-butylcarbamate or benzylcarbamate-protected α-amino acids by Staudinger reaction. [36] Cycloaddition was carried out with the ketenes derived from the mixed anhydride at −70 °C in dry tetrahydrofuran (**35**–**37**, Scheme 4). Again, the *cis* isomer was a major product but with lower yields, which could not be improved by changing the addition order of the reactants.

### 2.2. Deprotection of C3-NH_2_ Protecting Group

Since the phthalimide (Phth) moiety is the most commonly used amino protecting group in the ß-lactam ring cyclization reaction (because the cyclization of such Phth-protected ketenes proceeds in high yields), we wanted to optimize the conditions for its deprotection. However, the deprotection methods are quite harsh as they usually involve the use of a very strong base, such as hydrazine hydrate. A variety of Phth deprotection reagents were surveyed, including ethylenediamine, ethanolamine, methylhydrazine, and hydrazine hydrate. [37] The highest product isolated yield was obtained when hydrazine hydrate was used (in contrast, the yield was considerably lower with ethylenediamine and ethanolamine, which are also milder reagents). In the case of methyl hydrazine, the reaction was very slow, even with a high excess of reagent used.

**Scheme 5:**
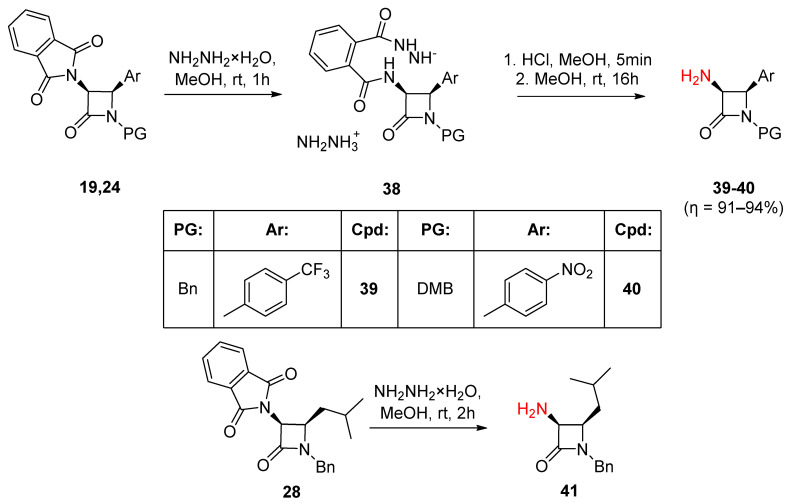
Deprotection of phthalimide protecting group with hydrazine hydrate. Reagents and reaction conditions: 1.NH_2_NH_2_ × H_2_O, MeOH, rt, 1–2 h; 2. HCl, MeOH, 5 min; 3. MeOH, rt, 16 h.

The problem, which has not been described in the literature, is that the reaction of the phthalimide-protected monocyclic ß-lactams (**39**–**40**) with the hydrazine hydrate very likely stops after 1 h because a salt forms with the hydrazine (**38**). Removal of the excess hydrazine and further addition of a few drops of concentrated hydrochloric acid breaks down the salt formed (Scheme 5). Once the HCl is removed, deprotection of the phthalimide group can continue, and the deprotected ß-lactams with the free amine group at C-3 can be isolated in high yields. Again, in the case of aliphatic substituents (**41**), which have an electron donor electronic effect than aromatic ones, deprotection with hydrazine hydrate proceeded rapidly and without any adjustments.

### 2.3. Deprotection of N1 Protecting Group

With the optimized conditions for the [2+2] cycloaddition in hand (Scheme 2), we moved our attention to the identification of the most optimal protecting group for the lactam amide nitrogen (i.e., N-1), that would (i) favor the cyclization, and (ii) be easily removable at the end. The preparation of target monocyclic ß-lactams was highly dependent on the success of N-1 deprotection. [38] The deprotection conditions had to be harsh enough to remove the protecting group without concurrent opening of the highly sensitive ß-lactam ring.

In the initial studies, we prepared a small set of *N*-benzyl ß-lactams (**19**, **23**, **32**–**33**) because we expected to be able to remove this protecting group easily with catalytic hydrogenation. Unfortunately, none of the traditional catalysts and hydrogen sources employed (e.g., Pd/C, Pd(OH)_2_ with cyclohexene) yielded any product. We have also attempted the aforementioned catalytic hydrogenation under elevated pressure (30 bar); LC-MS and NMR analyses of the reaction mixtures revealed that under these conditions, the N-1 benzyl group was cleaved, but the product yield was too low to enable the isolation and purification of the desired compounds (Table 1).

Since deprotection of the benzyl group proved highly problematic, we prepared some ß-lactams with dimethoxybenzyl protecting group at the N-1 position (**20**–**22**, **29**–**30**, **35**–**37**). There are several published procedures for removing the *para*-methoxybenzyl or di-methoxybenzyl group from the amide nitrogen. The procedures that we investigated are summarized in Table 1. First, we attempted to treat N-1-dimethoxybenzyl ß-lactam with strong acids, such as *p*-toluenesulfonic acid and trifluoroacetic acid (at 60 °C), but this yielded only starting material, and a side product that we assumed (based on NMR) was an opened ß-lactam ring [39]. Next, we have attempted to deprotect N-1 via oxidative cleavage of the dimethoxybenzyl protecting group. Several procedures using persulfate salts (e.g., potassium and/or ammonium persulfate, under various conditions including heating and acid addition [40,41]), which are known to be strong oxidizing agents, provided poor yields and numerous side products, making the isolation of the desired product by column chromatography extremely challenging.

**Scheme 6:**
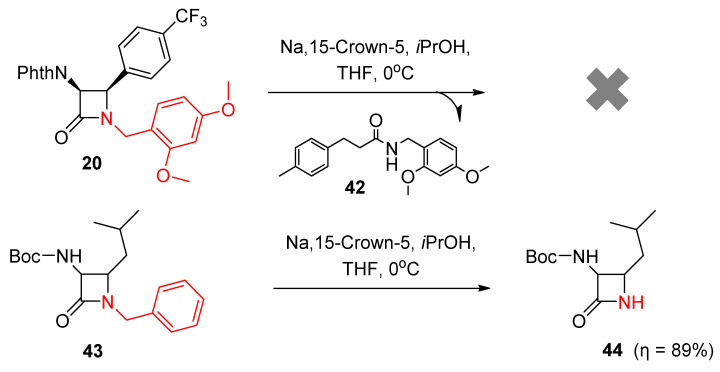
Deprotection of lactam nitrogen with ammonia-free Birch reduction. Reagents and reaction conditions: Na, 15-Crown-5, *i*PrOH, THF, 0 °C.

A process commonly used to deprotect lactam nitrogen in the literature was Birch reduction [42]. Since the standard process requires the use of toxic liquid ammonia and is often very time consuming, we turned our attention to the more recently published ammonia-free Birch reduction [43]. While the reaction under ammonia-free Birch reduction conditions provided no desired product in the case of dimethoxybenzyl, and benzyl ß-lactam derivates with aromatic C-4 substituents, an opened ß-lactam ring with eliminated phthalimide group has been isolated as an exclusive product. In the case of the trifluorophenyl group (**42**), the fluorine atoms were exchanged for hydrogen (Scheme 6). The situation was quite different for compounds with aliphatic substituents, where the above deprotection could be performed in excellent yields and with almost no side products detected (Table 1).

**Scheme 7:**
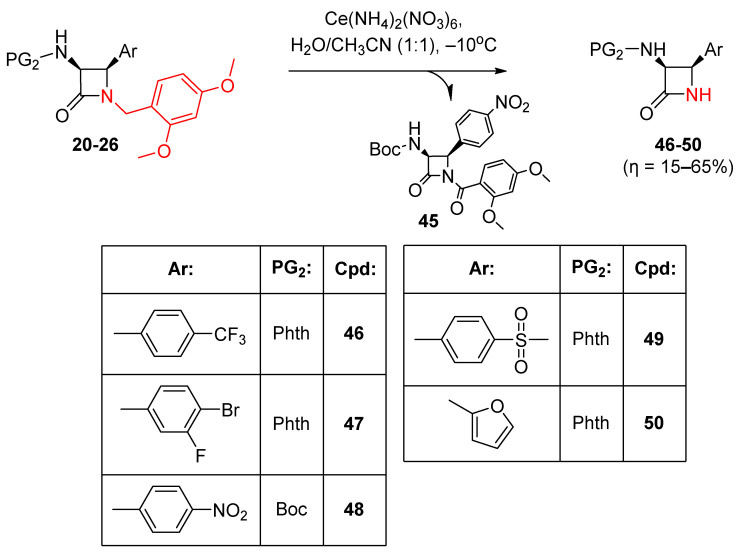
Deprotection of lactam nitrogen with cerium ammonium nitrate. Reagents and reaction conditions: Ce(NH_4_)_2_(NO_3_)_6_, H_2_O/CH_3_CN (1:1), −10 °C.

Finally, the best and most reliable deprotection approach was achieved by using a milder oxidant, cerium ammonium nitrate (**46**–**50**) [44]. Oxidative cleavage of *N*-dimethoxybenzyl protection with cerium ammonium nitrate in aqueous acetonitrile was achieved at the temperature of −10 °C, with the minimum formation of side product (<10%; e.g., compound **45**, Scheme 7). We found that the absence of atmospheric oxygen and the water/acetonitrile ratio were important factors in the amount of side product formed. Various relative amounts of acetonitrile/water were tried (from the ratio MeCN/H_2_O = 2:1 to 1:3), with the proportion of product varying from 14% to 52%. The best yield was obtained with a 1:1 ratio of water: acetonitrile, with minimal formation of oxidized, non-deprotected side products observed. However, oxidative dimethoxybenzyl cleavage with cerium ammonium nitrate was unsuccessful for monocyclic ß-lactams that had aliphatic substituents at the C-4 position.

**Scheme 8:**
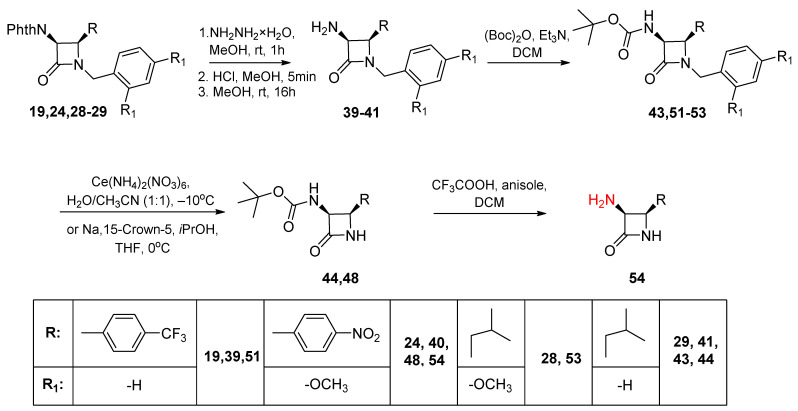
Synthesis of fully deprotected 3-amino-4-substituted azetidin-2-one **55.** Reagents and reaction conditions: 1. a. NH_2_NH_2_×H_2_O, MeOH, rt, 1 h; b. HCl, MeOH, 5 min; c. MeOH, rt, 16 h; 2. (Boc)_2_O, Et_3_N, DCM; 3. Ce(NH_4_)_2_(NO_3_)_6_, H_2_O/CH_3_CN (1:1), −10 °C or Na, 15-Crown-5, *i*PrOH, THF, 0 °C; 4 CF_3_COOH, anisole, DCM.

Finally, as an example of the synthetic potential of the methods described in this manuscript, we have prepared a fully deprotected 3-amino-4-substituted azetidin-2-one **54** (Scheme 8). The first step after cyclization was the cleavage of the phthalimide protecting group, as this requires the harshest conditions for deprotection, and the ß-lactam ring still protected at the lactam nitrogen is the most stable. Since oxidative cleavage of the dimethoxybenzyl protecting group with a free amino group at the C-3 position was not possible, we protected it again with a t-butyl carbamate protecting group that is stable to oxidation. For this purpose, we used di-*tert*-butyl dicarbonate with triethylamine in dichloromethane. After successful conversion of the phthalimide to the *t*-butyl carbamate protecting group (**43**, **51**–**53**, shown in Scheme 8), we used cerium ammonium nitrate to remove the dimethoxybenzyl protecting group from the ring nitrogen or ammonia-free Birch reduction in case of aliphatic substituent on C-4 position. Deprotection of the *t*-butyl carbamate protecting group with hydrochloric acid (4N HCl/dioxane) failed and resulted in the isolation of an opened monocyclic ß-lactam ring. However, the use of trifluoroacetic acid with anisole as a scavenger agent removed the Boc-protecting group in high yield (Scheme 8, compound **54**).

## 3. Conclusions

Using Staudinger [2+2] cycloaddition, we successfully synthesized a series of diprotected monocyclic ß-lactams with different substituents at the C-4 position. These initial ß-lactams had phthalimido-protected 3-amino group and dimethoxybenzyl protected ring nitrogen (N-1). Through an extensive study of previously published methods and their subsequent optimization, we have achieved the selective deprotection of both protecting groups in high yield. Oxidative cleavage with cerium ammonium nitrate selectively removed the N-1 protecting group when the aromatic substituents were at the C-4 position, while ammonia-free Birch reduction provided the highest yields for compounds with aliphatic C-4 substituents. For the removal of the phthalimido group, hydrazine hydrate provided the best yield, but in the case of aromatic substituents at the C-4 position, synthetic modification by HCl addition was required. The presented methods and the synthesized protected and partially deprotected 3-amino-4-substituted monocyclic ß-lactams are an important step toward new ß-lactams with potential pharmacological activities.

## 4. Materials and Methods

### 4.1. Chemistry and Chemical Characterization of Compounds

Unless otherwise stated, all reactions were carried out under argon atmosphere in flame-dried glassware. Chemicals and solvents were obtained from commercial sources (Sigma-Aldrich, Acros Organics, TCI Europe, fluorochem, and Apollo Sci) and were used as supplied. Dry solvents were prepared by distillation from CaH_2_ (CH_2_Cl_2_) or from a mixture of sodium and benzophenone (tetrahydrofuran). Other solvents (dimethylformamide, toluene, methanol, and CH_3_CN) were used directly from anhydrous Aldrich Sure/Seal bottles. Evaporation of the solvent was carried out under reduced pressure. Reactions were monitored by thin-layer chromatography (TLC) on silica gel aluminum plates (Merck DC Fertigplatten Kieselgel 60 GF254), visualized under UV light (254 nm), and stained with appropriate TLC stains for detection (ninhydrin, dinitrophenylhydrazine, and phospho-molybdic acid). The products were purified by flash column chromatography performed on Merck silica gel 60 (mesh size, 70–230) using the indicated solvents. Yields are reported for the purified products. ^1^H NMR and ^13^C NMR spectra were recorded at 295 K using a Bruker Avance III NMR spectrometer equipped with a Broadband decoupling inverse ^1^H probe, at 400 MHz and 100 MHz, respectively. Chemical shifts (δ) are given in parts per million (ppm) and refer to tetramethylsilane (TMS) as an internal standard. The coupling constants (*J*) are given in Hertz (Hz), and the splitting patterns are reported as: s, singlet; br s, broad singlet; d, doublet; dd, double doublet; t, triplet, and m, multiplet. Mass spectra were recorded using an ADVION Expres-sion CMSL mass spectrometer (Advion Inc., Ithaca, NY, USA). High-resolution, accurate mass measurements were performed using the ExactiveTM Plus Orbitrap mass spectrometer (Thermo Fisher Scientific Inc., Waltham, MA, USA).

### 4.2. General Procedure for the Synthesis of Schiff Bases (***1***–***17***)

To a solution of an appropriate aldehyde (1 EQ) in dry dichloromethane or dry methanol was added an amine (1 EQ). The resultant solution was stirred for 15 min before Na_2_SO_4_ (4 EQ) was added. The reaction mixture was then stirred at room temperature until TLC showed complete consumption of the starting material (30 min to 16 h). Next, the drying agent was removed by filtration, and the volatiles were removed under reduced pressure to afford the desired products, which were used in the next step without further purification.

*N*-Benzyl-1-(4-(trifluoromethyl)phenyl)methanimine (**1**), quantitative yield, brown oil. ^1^H NMR (400 MHz, DMSO-*d*_6_) δ 8.62 (s, 1H), 8.01 (d, *J* = 8.7 Hz, 2H), 7.83 (d, *J* = 8.7 Hz, 2H), 7.33 (m, 5H), 4.84 (s, 2H); Rf = 0.66 (EtOAc/Hexane = 1:1, *v*/*v*) as reported [45].

*N*-(2,4-Dimethoxybenzyl)-1-(4-(trifluoromethyl)phenyl)methanimine (**2**), quantitative yield, brown oil. ^1^H NMR (400 MHz, DMSO-*d*_6_) δ 8.50 (s, 1H), 7.97 (d, *J* = 8.0 Hz, 2H), 7.81 (d, *J* = 8.2 Hz, 2H), 7.16 (d, *J* = 8.3 Hz, 1H), 6.58 (d, *J* = 2.4 Hz, 1H), 6.51 (dd, *J* = 8.3, 2.4 Hz, 1H), 4.71 (s, 2H), 3.79 (s, *J* = 4.7 Hz, 3H), 3.76 (s, *J* = 3.6 Hz, 3H); Rf = 0.60 (EtOAc/Hexane = 1:1, *v*/*v*) as reported [45].

4-(((2,4-Dimethoxybenzyl)imino)methyl)benzonitrile (**3**), quantitative yield, colorless amorphous solid. ^1^H NMR (400 MHz, CDCl_3_) δ 8.33 (s, 1H), 7.89–7.79 (m, 2H), 7.74–7.61 (m, 2H), 7.21–7.13 (m, 1H), 6.53–6.42 (m, 2H), 4.80 (s, 2H), 3.81 (app s, 3H); Rf = 0.54 (EtOAc/Hexane = 1:1, *v*/*v*) as reported [45].

1-(3-Bromo-4-fluorophenyl)-*N*-(2,4-dimethoxybenzyl)methanimine (**4**), quantitative yield, yellow oil. ^1^H NMR (400 MHz, CDCl_3_) δ 8.21 (s, 1H), 8.00 (dd, *J* = 6.8, 2.1 Hz, 1H), 7.68–7.58 (m, 1H), 7.21–7.14 (m, 1H), 7.15–7.05 (m, 1H), 6.50–6.46 (m, 2H), 4.74 (s, 2H), 3.80 (app s, 6H). ^13^C NMR (100 MHz, CDCl_3_) δ 161.61, 160.32, 158.86, 158.37, 133.10, 130.33, 128.99, 128.91, 119.29, 116.64, 116.41, 104.16, 98.56, 58.78, 55.40, 55.40. HRMS (ESI+) m/z calc. for C_16_H_15_BrFNO_2_ 351.0270, found [M + H]^+^ 352.0338. Rf = 0.86 (EtOAc/Hexane = 1:1 *v*/*v*).

*N*-Benzyl-1-(4-nitrophenyl)methanimine (**5**), quantitative yield, yellow amorphous solid. ^1^H NMR (400 MHz, CDCl_3_) δ 8.46 (s, 1H), 8.26 (d, *J* = 8.7 Hz, 2H), 7.94 (d, *J* = 8.7 Hz, 2H), 7.43–7.15 (m, 5H), 4.88 (s, 2H); Rf = 0.66 (EtOAc/Hexane = 1:1, *v*/*v*) as reported [46].

*N*-(2,4-Dimethoxybenzyl)-1-(4-nitrophenyl)methanimine (**6**), quantitative yield, yellow amorphous solid. ^1^H NMR (400 MHz, DMSO-*d*_6_) δ 8.54 (s, 1H), 8.31–8.26 (m, 2H), 8.04–7.98 (m, 2H), 7.16 (d, *J* = 8.3 Hz, 1H), 6.58 (d, *J* = 2.4 Hz, 1H), 6.51 (dd, *J* = 8.3, 2.4 Hz, 1H), 4.73 (s, 2H), 3.79 (s, 3H), 3.76 (s, 3H); Rf = 0.46 (EtOAc/Hexane = 1:1, *v*/*v*) as reported [47].

*N*-(2,4-Dimethoxybenzyl)-1-(4-(methylsulfonyl)phenyl)methanimine (**7**), quantitative yield, pale yellow amorphous solid. ^1^H NMR (400 MHz, DMSO-*d*_6_) δ 8.51 (s, 1H), 8.00 (m, 4H), 7.16 (d, *J* = 8.3 Hz, 1H), 6.58 (d, *J* = 2.4 Hz, 1H), 6.51 (dd, *J* = 8.3, 2.4 Hz, 1H), 4.72 (s, 2H), 3.79 (s, 3H), 3.76 (s, 3H), 3.25 (s, 3H). ^13^C NMR (100 MHz, CDCl_3_) δ 160.40, 159.59, 158.40, 141.76, 141.24, 130.37, 128.93, 127.63, 119.01, 104.21, 98.57, 59.13, 55.41, 44.46. HRMS (ESI+) m/z calc. for C_17_H_19_NO_4_S 333.1035, found [M + H]^+^ 334.1104. Rf = 0.25 (EtOAc:Hex = 1:1, *v*/*v*).

4-(((2,4-Dimethoxybenzyl)imino)methyl)-*N,N*-dimethylaniline (**8**), quantitative yield, colorless amorphous solid. ^1^H NMR (400 MHz, CDCl_3_) δ 8.19 (d, *J* = 1.4 Hz, 1H), 7.63 (d, *J* = 8.9 Hz, 2H), 7.18 (d, *J* = 8.9 Hz, 2H), 6.68 (d, *J* = 8.9 Hz, 1H), 6.48–6.42 (m, 2H), 4.68 (s, 2H), 3.79 (s, 3H), 3.78 (s, 3H), 2.98 (s, 6H). ^13^C NMR (100 MHz, CDCl_3_) δ 162.19, 159.96, 158.22, 152.06, 129.96, 129.70, 124.44, 120.58, 111.61, 111.01, 104.01, 98.42, 58.71, 55.37, 50.39, 40.21, 40.06. HRMS (ESI+) m/z calc. for C_18_H_22_N_2_O_2_ 298.1681, found [M + H]^+^ 299.1751; Rf = 0.63 (EtOAc/Hexane = 1:1, *v*/*v*).

*N*-Benzyl-1-phenylmethanimine (**9**), quantitative yield, brown oil. ^1^H NMR (400 MHz, DMSO-*d*_6_) δ 8.49 (s, 1H), 7.84–7.75 (m, 2H), 7.50–7.22 (m, 8H), 4.77 (s, *J* = 1.2 Hz, 2H); Rf = 0.65 (EtOAc/Hex = 1:1 *v*/*v*) as reported [48].

*N*-(2,4-Dimethoxybenzyl)-1-(furan-2-yl)methanimine (**10**), quantitative yield, dark brown oil. ^1^H NMR (400 MHz, CDCl_3_) δ 8.08 (s, 1H), 7.49 (s, 1H), 7.21–7.16 (m, 1H), 6.73 (d, *J* = 3.4 Hz, 1H), 6.49–6.44 (m, 3H), 4.74 (s, 2H), 3.80 (s, 3H), 3.79 (s, 3H); Rf = 0.36 (EtOAc/Hexane = 1:1, *v*/*v*) as reported [47].

*N*-(2,4-Dimethoxybenzyl)-1-(1*H*-imidazol-5-yl)methanimine (**11**), quantitative yield, colorless amorphous solid. ^1^H NMR (400 MHz, DMSO-*d*_6_) δ 8.22 (s, 1H), 7.72–7.69 (m, 1H), 7.41 (s, 1H), 7.13 (d, *J* = 8.3 Hz, 1H), 6.56 (d, *J* = 2.4 Hz, 1H), 6.49 (dd, *J* = 8.3, 2.4 Hz, 1H), 4.57 (s, 2H), 3.77 (s, 3H), 3.75 (s, 3H). ^13^C NMR (100 MHz, DMSO-*d*_6_) δ 160.21, 158.35, 130.56, 120.00, 104.92, 98.69, 58.39, 55.82, 55.63. HRMS (ESI+) m/z calc. for C_13_H_15_N_3_O_2_ 245.1164, found [M + H]^+^ 246.1234. Rf = 0.1 (EtOAc).

1-(Benzo[*b*]thiophen-2-yl)-*N*-benzylmethanimine (**12**), quantitative yield, yellow amorphous solid. ^1^H NMR (400 MHz, DMSO-*d*_6_) δ 8.76 (s, 1H), 7.98–7.8 (m 2H), 7.87 (s, 1H), 7.44–7.40 (m, 2H), 7.38–7.21 (m, 5H), 4.80 (s, 2H); Rf = 0.67 (EtOAc/Hexane = 1:1, *v*/*v*) as reported [49].

1-(Benzo[*d*][1,3]dioxol-5-yl)-*N*-benzylmethanimine (**13**), quantitative yield, colorless amorphous solid. ^1^H NMR (400 MHz, CDCl_3_) δ 8.26 (t, *J* = 1.4 Hz, 1H), 7.41 (d, *J* = 1.4 Hz, 1H), 7.35–7.22 (m, 5H), 7.14 (dd, *J* = 8.0, 1.6 Hz, 1H), 6.82 (d, *J* = 7.9 Hz, 1H), 5.98 (s, 2H), 4.77 (s, 2H); Rf = 0.60 (EtOAc/Hexane = 1:1, *v*/*v*) as reported [50].

1-(Benzo[d][1,3]dioxol-5-yl)-*N*-(2,4-dimethoxybenzyl)methanimine (**14**), quantitative yield, colorless amorphous solid. ^1^H NMR (400 MHz, DMSO-*d*_6_) δ 8.27 (s, 1H), 7.30 (d, *J* = 1.5 Hz, 1H), 7.20 (dd, *J* = 7.9, 1.5 Hz, 1H), 7.12 (d, *J* = 8.3 Hz, 1H), 6.97 (d, *J* = 8.0 Hz, 1H), 6.56 (d, *J* = 2.4 Hz, 1H), 6.49 (dd, *J* = 8.3, 2.4 Hz, 1H), 6.07 (s, 2H), 4.60 (s, 2H), 3.78 (s, 3H), 3.75 (s, 3H); Rf = 0.65 (EtOAc/Hexane = 1:1, *v*/*v*) as reported [50].

*N*-Benzyl-3-methylbutan-1-imine (**15**), quantitative yield, light orange oil. ^1^H NMR (400 MHz, CDCl_3_) δ 7.38–7.25 (m, 5H), 6.32 (s, 1H), 3.90 (s, 2H), 2.03–1.81 (m, 3H), 0.99–0.76 (m, 6H). ^13^C NMR (100 MHz, CDCl_3_) δ 179.39, 138.07, 128.76, 127.95, 127.81, 45.70, 44.53, 25.90, 22.58. HRMS (ESI+) m/z calc. for C_12_H_17_N 175.1361, found [M + H]^+^ 176.1435. Rf = 0.65 (EtOAc/Hex = 1:1, *v*/*v*).

*N*-(2,4-Dimethoxybenzyl)-3-methylbutan-1-imine (**16**), quantitative yield, yellow oil. ^1^H NMR (400 MHz, CDCl_3_) δ 7.15 (d, *J* = 8.1 Hz, 1H), 6.73 (s, 1H), 6.46–6.38 (m, 2H), 3.86 (s, 2H), 3.81 (s, 3H), 3.79 (s, 3H), 2.00–1.88 (m, 3H), 0.88 (s, 3H), 0.87 (s, 3H). ^13^C NMR (100 MHz, CDCl_3_) δ 179.20, 160.92, 158.55, 130.46, 118.49, 104.03, 98.55, 55.38, 55.29, 45.95, 40.20, 25.93, 22.64. Rf = 0.63 (EtOAc/Hex = 1:1, *v*/*v*).

*N*-(2,4-Dimethoxybenzyl)heptan-1-imine (**17**), quantitative yield, orange oil. ^1^H NMR (400 MHz, CDCl_3_) δ 9.48 (s, 1H), 7.18–7.15 (m, 1H), 6.46–6.39 (m, 2H), 3.92 (s, 2H), 3.84–3.73 (m, 6H), 1.93–1.79 (m, 4H), 1.51–1.20 (m, 6H), 0.95–0.82 (m, 3H). ^13^C NMR (100 MHz, CDCl_3_) δ 176.84, 161.52, 158.65, 131.30, 114.65, 104.24, 98.38, 55.32, 55.27, 39.00, 22.73, 14.03. HRMS (ESI+) m/z calc. for C_16_H_25_NO_2_ 263.1885, found [M + H]^+^ 264.1954. Rf = 0.85 (EtOAc/Hex = 1:1, *v*/*v*).

### 4.3. General Procedures for the Synthesis of Ketene Precursors

#### 4.3.1. General Procedure for the Synthesis of Acid Chloride (**18**)

*N*-phthaloylglycine (2.00 g, 9.75 mmol, 1 EQ) was dissolved in dry dichloromethane (10 mL), and the solution was cooled to 0 °C using an ice bath before oxalyl chloride (0.95 mL, 10.73 mmol, 1.1 EQ) was added dropwise over 30 min. Upon complete addition, the reaction mixture was stirred at 0 °C for an additional 2 h, and the solvent was removed under reduced pressure without heating. The acyl chlorides thus obtained were used in the subsequent step without further purification.

2-(1,3-Dioxoisoindolin-2-yl)acetyl chloride (**18**), quantitative yield, yellow amorphous solid. ^1^H NMR (400 MHz, CDCl_3_) 7.95–7.89 (m, 1H), 7.82–7.76 (m, 1H), 4.83 (s, 1H); as reported [51].

#### 4.3.2. General Procedure for the Synthesis of Diazoketone (**31**)

*N*-Benzyloxycarbonylglycine (2.09 g, 10.0 mmol, 1 EQ) was dissolved in dry tetrahydrofuran (20 mL), and the resultant solution was cooled to −20 °C using a sodium chloride ice bath before triethylamine (1.39 mL, 10.0 mmol, 1 EQ) was added in one portion. Ethyl chloroformate (1.91 mL, 10.0 mmol, 1 EQ) was then added dropwise, and the reaction mixture was stirred for another 1 h. The white precipitate formed was removed by filtration. To the filtrate were slowly added dry acetonitrile (80 mL) (4:1 solution in THF) and (trimethylsilyl)diazomethane (2.0 M solution in hexane, 10 mL, 20.0 mmol, 2 EQ). The resultant reaction mixture was then stirred at 4 °C for 24–48 h. The reaction was quenched by the addition of diethyl ether and 10% (m/m) aqueous citric acid. The organic phase was then washed with saturated aqueous NaHCO_3_ and brine. The organic layer was dried over Na_2_SO_4,_ and the solvents were evaporated. The diazoketone was purified by silica gel column chromatography using EtOAc:Hex = 1:1, *v*/*v* as eluent.

Benzyl (3-diazo-2-oxopropyl)carbamate (**31**), quantitative yield, transparent amorphous solid. ^1^H NMR (400 MHz, CDCl_3_) δ (ppm) = 7.42–7.31 (m, 5H), 5.46 (s, 1H), 5.39 (s, 1H), 5.13 (s, 2H), 3.99 (s, 1H); as reported [52].

#### 4.3.3. General Procedure for the Synthesis of Mixed Anhydride

In a flame-dried flask, *N*-(*tert*-butoxycarbonyl)glycine (3.00 g, 17.13 mmol, 1 EQ) was dissolved in dry tetrahydrofuran (20 mL) and placed under an argon atmosphere. The solution was cooled to −60 °C, and triethylamine (2.62 mL, 18.84 mmol, 1.1 EQ) was added in one portion. Then ethyl chloroformate (2.13 mL, 22.27 mmol, 1.3EQ) was added dropwise over a period of 30 min. After the complete addition of the reagent, the reaction mixture was stirred at −40 °C for another 2 h. The resultant reaction mixture was then directly used in the next step without any further purification. The same reaction conditions were used for the synthesis of 2-(((benzyloxy)carbonyl)amino)acetic anhydride from ((benzyloxy)carbonyl)glycine.

### 4.4. General Procedure for the Synthesis of Monocyclic Beta Lactam Core I (***19***–***30***)

Schiff base (1 EQ) was dissolved in dry toluene (0.1–0.2 mmol/mL) in a flame-dried flask and placed under an argon atmosphere. Triethylamine (2.5 EQ) was then added in one portion, and the resultant solution was heated to 80 °C, before 2-(1,3-dioxoisoindolin-2-yl)acetyl chloride (1.3 EQ), dissolved in in dry toluene, was added dropwise over a period of 30 min. Upon complete addition, the reaction was stirred at 80 °C for a further 1.5–3.5 h. The reaction mixture was then cooled to room temperature, and the volatiles were removed in vacuo. The solid residue thus obtained was redissolved in ethyl acetate. The organic phase was washed with 10% aq. citric acid solution, saturated NaHCO_3,_ and brine. The organic phase was dried (Na_2_SO_4_), filtered, then concentrated in vacuo. Some cyclized ß-lactams were purified by silica gel column chromatography using EtOAc: Hex as eluent.

2-(1-Benzyl-2-oxo-4-(4-(trifluoromethyl)phenyl)azetidin-3-yl)isoindoline-1,3-dione (**19**), yield: 51%, colorless amorphous solid. The reaction was carried out according to General Procedure I using *N*-benzyl-1-(4-(trifluoromethyl)phenyl)methanimine (**1**), (1.32 g, 5 mmol, 1.0 EQ), triethylamine (1.74 mL, 12.5 mmol, 2.5 EQ) and 2-(1,3-dioxoisoindolin-2-yl)acetyl chloride (1.45 g, 6.5 mmol, 1.3 EQ). Product was purified by silica gel column chromatography using EtOAc: n-Hex = 2:1 (*v*/*v*) as eluent. ^1^H NMR (400 MHz, DMSO-*d*_6_) δ 7.80–7.70 (m, 4H), 7.51 (d, *J* = 8.2 Hz, 2H), 7.39–7.28 (m, 7H), 5.77 (d, *J* = 5.4 Hz, 1H), 5.18 (d, *J* = 5.4 Hz, 1H), 4.82 (d, *J* = 15.4 Hz, 1H), 4.47 (d, *J* = 15.4 Hz, 1H). ^13^C NMR (100 MHz, CDCl_3_) δ 166.73, 163.56, 137.29, 134.53, 134.42, 131.04, 129.09, 128.64, 128.26, 127.79, 125.50, 125.47, 123.59, 60.16, 59.84, 45.83. HRMS (ESI+) m/z calc. for C_25_H_17_F_3_N_2_O_3_ 450.1191, found [M + H]^+^ 451.1260. Rf = 0.42 (EtOAc/n-Hex; 2:1, *v*/*v*).

2-(1-(2,4-Dimethoxybenzyl)-2-oxo-4-(4-(trifluoromethyl)phenyl)azetidin-3-yl)isoindoline-1,3-dione (**20**), yield: 55%, light brown amorphous solid. The reaction was carried out according to General Procedure I using *N*-(2,4-dimethoxybenzyl)-1-(4-(trifluoromethyl)phenyl)methanimine (**2**), (3.23 g, 10 mmol, 1.0 EQ), triethylamine (3.48 mL, 25 mmol, 2.5 EQ) and 2-(1,3-dioxoisoindolin-2-yl)acetyl chloride (**18**) (2.91 g, 13 mmol, 1.3 EQ). Product was purified by silica gel column chromatography using EtOAc: n-Hex = 1:1 (*v*/*v*) as eluent. ^1^H NMR (400 MHz, CDCl_3_) δ 7.71–7.60 (m, 4H), 7.44 (d, *J* = 8.2 Hz, 2H), 7.33 (d, *J* = 8.1 Hz, 2H), 7.15 (d, *J* = 8.3 Hz, 1H), 6.43 (dd, *J* = 8.3, 2.4 Hz, 1H), 6.37 (d, *J* = 2.3 Hz, 1H), 5.46 (d, *J* = 5.4 Hz, 1H), 4.90 (d, *J* = 14.3 Hz, 1H), 4.84 (d, *J* = 5.4 Hz, 1H), 4.30 (d, *J* = 14.3 Hz, 1H), 3.79 (s, 3H), 3.56 (s, 3H). ^13^C NMR (100 MHz, CDCl_3_) δ 166.77, 163.44, 161.18, 158.59, 138.33, 134.33, 131.59, 131.07, 127.60, 125.17, 125.13, 123.49, 115.03, 104.34, 98.33, 60.65, 59.54, 55.41, 55.00, 40.82. HRMS (ESI+) m/z calc. for C_27_H_21_F_3_N_2_O_5_ 510.1403, found [M + H]^+^ 511.1470. Rf = 0.31 (EtOAc/n-Hex; 1:1, *v*/*v*).

4-(1-(2,4-Dimethoxybenzyl)-3-(1,3-dioxoisoindolin-2-yl)-4-oxoazetidin-2-yl)benzonitrile (**21**), yield: 48%, colorless amorphous solid. The reaction was carried out according to General Procedure I using 4-(((2,4-dimethoxybenzyl)imino)methyl)benzonitrile (**3**) (0.32 g, 1.15 mmol, 1 EQ), triethylamine (0.40 mL, 2.87 mmol, 2.5 EQ) and 2-(1,3-dioxoisoindolin-2-yl)acetyl chloride (**18**) (0.33 g, 1.50 mmol, 1.3 EQ). Product was purified by silica gel column chromatography using EtOAc: n-Hex = 2:1 (*v*/*v*) as eluent. ^1^H NMR (400 MHz, DMSO-*d*_6_) δ 7.80–7.72 (m, 4H), 7.63 (d, *J* = 8.4 Hz, 2H), 7.28 (d, *J* = 8.2 Hz, 2H), 7.21–7.17 (m, 1H), 6.49–6.46 (m, 2H), 5.58 (d, *J* = 5.5 Hz, 1H), 5.01 (d, *J* = 5.4 Hz, 1H), 4.61 (d, *J* = 14.5 Hz, 1H), 4.34 (d, *J* = 14.6 Hz, 1H), 3.74 (s, 3H), 3.57 (s, 3H). ^13^C NMR (100 MHz, CDCl_3_) δ = 166.69, 163.32, 161.26, 158.57, 139.89, 134.49, 131.99, 131.65, 130.97, 127.88, 123.58, 118.41, 114.87, 111.87, 104.39, 98.39, 60.72, 59.57, 55.44, 55.02, 40.97. MS (ESI+, m/z), 468.4 ([M + H]^+^). Rf = 0.24 (EtOAc/n-Hex; 1:1, *v*/*v*).

2-(2-(4-Bromo-3-fluorophenyl)-1-(2,4-dimethoxybenzyl)-4-oxoazetidin-3-yl)isoindoline-1,3-dione (**22**), yield: 51%, pale yellow amorphous solid. The reaction was carried out according to General Procedure I using 1-(3-bromo-4-fluorophenyl)-*N*-(2,4-dimethoxybenzyl)methanimine (**4**) (0.53 g, 1.5 mmol, 1 EQ), triethylamine (0.52 mL, 3.75 mmol, 2.5 EQ) and 2-(1,3-dioxoisoindolin-2-yl)acetyl chloride (**18**) (0.44 g, 1.95 mmol, 1.3 EQ). Product was purified by silica gel column chromatography using EtOAc: n-Hex = 2:1 (*v*/*v*) as eluent. ^1^H NMR (400 MHz, CDCl_3_) δ 7.73–7.64 (m, 4H), 7.37 (dd, *J* = 6.5, 2.2 Hz, 1H), 7.17–7.10 (m, 2H), 6.91 (t, *J* = 8.4 Hz, 1H), 6.43 (dd, *J* = 8.3, 2.4 Hz, 1H), 6.37 (d, *J* = 2.3 Hz, 1H), 5.40 (d, *J* = 5.3 Hz, 1H), 4.82 (d, *J* = 14.3 Hz, 1H), 4.74 (d, *J* = 5.4 Hz, 1H), 4.30 (d, *J* = 14.3 Hz, 1H), 3.80 (s, 3H), 3.61 (s, 3H). ^13^C NMR (100 MHz, CDCl_3_) δ 166.77, 163.36, 161.19, 158.57, 134.36, 132.52, 131.60, 131.51, 131.47, 131.14, 127.91, 127.84, 123.55, 116.33, 116.10, 115.03, 104.37, 98.37, 60.22, 59.61, 55.43, 55.10, 40.71. HRMS (ESI+) m/z calc. for C_26_H_20_BrFN_2_O_5_ 538.0540, found [M + H]^+^ 539.0606. Rf = 0.56 (EtOAc/n-Hex; 2:1, *v*/*v*).

2-(1-Benzyl-2-(4-nitrophenyl)-4-oxoazetidin-3-yl)isoindoline-1,3-dione (**23**), yield: 47%, pale yellow amorphous solid. The reaction was carried out according to General Procedure I using *N*-benzyl-1-(4-nitrophenyl)methanimine (**5**) (2.10 g, 10 mmol, 1 EQ), triethylamine (3.48 mL, 25 mmol, 2.5 EQ) and 2-(1,3-dioxoisoindolin-2-yl)acetyl chloride (**18**) (2.91 g, 13 mmol, 1.3 EQ). Product was purified by silica gel column chromatography using EtOAc: n-Hex = 2:1 (*v*/*v*) as eluent. ^1^H NMR (400 MHz, CDCl_3_) δ 8.05 (d, *J* = 8.8 Hz, 2H), 7.74–7.60 (m, 4H), 7.39 (d, *J* = 8.7 Hz, 2H), 7.36–7.22 (m, 5H), 5.57 (d, *J* = 5.5 Hz, 1H), 5.06 (d, *J* = 14.8 Hz, 1H), 4.93 (d, *J* = 5.4 Hz, 1H), 4.26 (d, *J* = 14.8 Hz, 1H). ^13^C NMR (100 MHz, CDCl_3_) δ 166.64, 163.40, 147.84, 140.76, 134.59, 134.35, 130.93, 129.16, 128.66, 128.40, 128.33, 123.72, 123.70, 60.16, 59.88, 46.08. HRMS (ESI+) m/z calc. for C_24_H_17_N_3_O_5_ 427.1168, found [M + H]^+^ 433.1386. Rf = 0.25 (EtOAc/n-Hex; 1:1, *v*/*v*).

2-(1-(2,4-Dimethoxybenzyl)-2-(4-nitrophenyl)-4-oxoazetidin-3-yl)isoindoline-1,3-dione (**24**), yield: 51%, pale yellow amorphous solid. The reaction was carried out according to General Procedure I using *N*-(2,4-dimethoxybenzyl)-1-(4-nitrophenyl)methanimine (**6**) (3.00 g, 10 mmol, 1 EQ), triethylamine (3.48 mL, 25 mmol, 2.5 EQ) and 2-(1,3-dioxoisoindolin-2-yl)acetyl chloride (**18**) (2.91 g, 13 mmol, 1.3 EQ). Product was purified by silica gel column chromatography using EtOAc: n-Hex = 2:1 (*v*/*v*) as eluent. ^1^H NMR (400 MHz, CDCl_3_) δ 8.04 (d, *J* = 8.8 Hz, 2H), 7.72–7.60 (m 4H), 7.39 (d, *J* = 8.6 Hz, 2H), 7.16 (d, *J* = 8.3 Hz, 1H), 6.43 (dd, *J* = 8.3, 2.3 Hz, 1H), 6.36 (d, *J* = 2.3 Hz, 1H), 5.49 (d, *J* = 5.5 Hz, 1H), 4.91 (s, *J* = 14.3 Hz, 1H), 4.88 (d, *J* = 5.3 Hz, 1H), 4.33 (d, *J* = 14.3 Hz, 1H), 3.79 (s, 3H), 3.56 (s, 3H). ^13^C NMR (100 MHz, CDCl_3_) δ 166.67, 163.28, 161.29, 158.57, 147.57, 141.91, 134.50, 131.66, 130.96, 128.10, 123.64, 123.42, 114.82, 104.43, 98.41, 60.58, 59.58, 55.44, 55.06, 41.02. HRMS (ESI+) m/z calc. for C_26_H_21_N_3_O_7_ 487.1380, found [M + H]^+^ 488.1443. Rf = 0.42 (EtOAc/n-Hex; 2:1, *v*/*v*).

2-(1-(2,4-Dimethoxybenzyl)-2-(4-(methylsulfonyl)phenyl)-4-oxoazetidin-3-yl)isoindoline-1,3-dione (**25**), yield: 45%, pale yellow amorphous solid. The reaction was carried out according to General Procedure I using *N*-(2,4-dimethoxybenzyl)-1-(4-(methylsulfonyl)phenyl)methanimine (**7**) (1.00 g, 3.00 mmol, 1 EQ), triethylamine (1.04 mL, 7.50 mmol, 2.5 EQ) and 2-(1,3-dioxoisoindolin-2-yl)acetyl chloride (**18**) (0.87 g, 3.90 mmol, 1.3 EQ). Product was purified by silica gel column chromatography using EtOAc: n-Hex = 2:1 (*v*/*v*) as eluent. ^1^H NMR (400 MHz, DMSO-*d*_6_) δ 7.86–7.69 (m, 4H), 7.68 (d, *J* = 8.2 Hz, 2H), 7.35 (d, *J* = 8.3 Hz, 2H), 7.20 (d, *J* = 8.8 Hz, 1H), 6.51–6.45 (m, 2H), 5.59 (d, *J* = 5.5 Hz, 1H), 5.03 (d, *J* = 5.4 Hz, 1H), 4.64 (d, *J* = 14.5 Hz, 1H), 4.34 (d, *J* = 14.6 Hz, 1H), 3.74 (s, 3H), 3.58 (s, 3H), 2.98 (s, 3H). ^13^C NMR (100 MHz, DMSO-*d*_6_) δ 166.80, 163.35, 161.07, 158.68, 141.06, 140.45, 135.42, 131.54, 130.78, 128.35, 126.90, 123.79, 115.39, 105.13, 98.72, 60.68, 59.76, 55.69, 55.64, 43.71, 41.04. HRMS (ESI+) m/z calc. for C_27_H_24_N_2_O_7_S 520.1304, found [M + H]^+^ 521.1378. Rf = 0.36 (EtOAc/n-Hex; 2:1, *v*/*v*).

2-(1-(2,4-Dimethoxybenzyl)-2-(furan-2-yl)-4-oxoazetidin-3-yl)isoindoline-1,3-dione (**26**), yield: 51%, colorless amorphous solid. The reaction was carried out according to General Procedure I using *N*-(2,4-dimethoxybenzyl)-1-(furan-2-yl)methanimine (**10**) (1.23 g, 5 mmol, 1 EQ), triethylamine (1.74 mL, 12.5 mmol, 2.5 EQ) and 2-(1,3-dioxoisoindolin-2-yl)acetyl chloride (**18**) (1.45 g, 6.5 mmol, 1.3 EQ). Product was purified by silica gel column chromatography using EtOAc: n-Hex = 2:1 (*v*/*v*) as eluent. ^1^H NMR (400 MHz, CDCl_3_) δ 7.81–7.63 (m, 4H), 7.21–7.18 (m, 1H), 7.15 (d, *J* = 8.1 Hz, 1H), 6.48–6.40 (m, 2H), 6.29 (d, *J* = 3.3 Hz, 1H), 6.18 (dd, *J* = 3.3, 1.8 Hz, 1H), 5.42 (d, *J* = 5.0 Hz, 1H), 4.84 (d, *J* = 5.0 Hz, 1H), 4.80 (d, *J* = 14.5 Hz, 1H), 4.22 (d, *J* = 14.5 Hz, 1H), 3.80 (s, 3H), 3.71 (s, 3H). ^13^C NMR (100 MHz, CDCl_3_) δ 166.73, 163.17, 161.01, 158.61, 148.22, 142.85, 134.20, 131.45, 131.38, 123.51, 115.39, 110.56, 109.87, 104.20, 98.41, 59.03, 55.41, 55.29, 55.12, 40.18. HRMS (ESI+) m/z calc. for C_24_H_20_N_2_O_6_ 432.1321, found [M + H]^+^ 433.1386. Rf = 0.23 (EtOAc/n-Hex; 2:1, *v*/*v*).

2-(1-(2,4-Dimethoxybenzyl)-2-(1*H*-imidazol-5-yl)-4-oxoazetidin-3-yl)isoindoline-1,3-dione (**27**), yield: 33%, light brown amorphous solid. The reaction was carried out according to General Procedure I using *N*-(2,4-dimethoxybenzyl)-1-(1*H*-imidazol-5-yl)methanimine (**11**) (1.19 g, 7.7 mmol, 1 EQ), triethylamine (1.35 mL, 15.4 mmol, 2 EQ) and 2-(1,3-dioxoisoindolin-2-yl)acetyl chloride (**18**) (2.23 g, 10 mmol, 1.3EQ). Product was purified by silica gel column chromatography using DKM: MeOH = 15:1 (*v*/*v*) as eluent. ^1^H NMR (400 MHz, CDCl_3_) δ 9.69 (s, 1H), 7.90–7.79 (m, 4H), 7.57–7.47 (m, 1H), 6.85 (d, *J* = 8.2 Hz, 1H), 6.38–6.23 (m, 2H), 4.92 (d, *J* = 16.7 Hz, 1H), 4.78 (d*, J* = 7.5 Hz, 2H), 4.74 (d, *J* = 7.3 Hz, 1H), 4.62 (d, *J* = 16.6 Hz, 1H), 3.81 (s, 3H), 3.75 (s, 3H). ^13^C NMR (100 MHz, CDCl_3_) δ 185.51, 184.92, 167.93, 161.04, 158.25, 140.99, 139.31, 136.01, 134.21, 132.14, 123.59, 104.24, 98.57, 63.70, 55.41, 55.38, 55.21, 39.94. HRMS (ESI+) m/z calc. for C_23_H_20_N_4_O_5_ 432.1434, found [M + H]^+^ 433.1500. Rf = 0.42 (DKM/MeOH; 9:1, *v*/*v*).

2-(1-Benzyl-2-isobutyl-4-oxoazetidin-3-yl)isoindoline-1,3-dione (**28**), yield: 31%, colorless amorphous solid. The reaction was carried out according to General Procedure I using *N*-Benzyl-3-methylbutan-1-imine (**15**) (2 g, 11.6 mmol, 1 EQ), triethylamine (3.21 mL, 23 mmol, 2 EQ) and 2-(1,3-dioxoisoindolin-2-yl)acetyl chloride (**18**) (3.1 g, 15 mmol, 1.3 EQ). Product was purified by silica gel column chromatography using EtOAc: n-Hex = 1:1 (*v*/*v*) as eluent. ^1^H NMR (400 MHz, CDCl_3_) δ 7.95–7.66 (m, 4H), 7.34–7.14 (m, 5H), 6.41 (d, *J* = 13.8 Hz, 1H), 5.26 (dd, *J* = 13.8, 7.3 Hz, 1H), 4.84–4.44 (m, 4H), 2.38–2.22 (m, 1H), 0.98 (d, *J* = 6.7 Hz, 3H), 0.93 (d, *J* = 6.7 Hz, 3H). ^13^C NMR (100 MHz, CDCl_3_) δ 167.98, 164.64, 136.60, 134.07, 132.27, 128.52, 127.37, 125.63, 123.56, 60.38, 48.50, 39.81, 29.57, 22.85, 22.68. HRMS (ESI+) m/z calc. for C_22_H_22_N_2_O_3_ 362.1630, found [M + H]^+^ 363.1694. Rf = 0.54 (EtOAc/n-Hex; 1:1, *v*/*v*).

2-(1-(2,4-Dimethoxybenzyl)-2-isobutyl-4-oxoazetidin-3-yl)isoindoline-1,3-dione (**29**), yield: 25%, colorless amorphous solid. The reaction was carried out according to General Procedure I using *N*-(2,4-Dimethoxybenzyl)-3-methylbutan-1-imine (**16**) (2.73 g, 11.6 mmol, 1 EQ), triethylamine (3.21 mL, 23 mmol, 2 EQ) and 2-(1,3-dioxoisoindolin-2-yl)acetyl chloride (**18**) (3.36 g, 15 mmol, 1.3 EQ). Product was purified by silica gel column chromatography using EtOAc: n-Hex = 1:1 (*v*/*v*) as eluent. ^1^H NMR (400 MHz, DMSO-*d*_6_) δ 7.99–7.82 (m, 4H), 7.01–6.93 (m, 1H), 6.84–6.74 (m, 1H), 6.63–6.51 (m, 1H), 6.50–6.44 (m, 1H), 5.14–5.00 (m, 1H), 4.83–4.55 (m, 4H), 3.90–3.66 (m, 6H), 2.37–2.13 (m, 1H), 0.95 (d, *J* = 6.7 Hz, 3H), 0.88 (d, *J* = 6.7 Hz, 3H). ^13^C NMR (100 MHz, DMSO-*d*_6_) δ 168.00, 165.35, 160.05, 157.83, 135.21, 132.07, 127.88, 123.80, 120.61, 116.68, 105.05, 98.63, 65.35, 55.86, 55.62, 43.67, 41.94, 29.24, 23.36. HRMS (ESI+) m/z calc. for C_24_H_26_N_2_O_5_ 422.1842, found [M + H]^+^ 423.1910. Rf = 0.45 (EtOAc/n-Hex; 1:1, *v*/*v*).

2-(1-(2,4-Dimethoxybenzyl)-2-hexyl-4-oxoazetidin-3-yl)isoindoline-1,3-dione (**30**), yield: 11%, brown oil. The reaction was carried out according to General Procedure I using *N*-(2,4-Dimethoxybenzyl)heptan-1-imine (**17**) (2.31 g, 8.76 mmol, 1EQ), triethylamine (3.05 mL, 21.9 mmol, 2.5 EQ) and 2-(1,3-dioxoisoindolin-2-yl)acetyl chloride (**18**) (2.53 g, 11.3 mmol, 1.3 EQ). Product was purified by silica gel column chromatography using EtOAc: n-Hex = 1:1 (*v*/*v*) as eluent. 1H NMR (400 MHz, CDCl_3_) δ 7.93–7.66 (m, 4H), 7.07–6.94 (m, 1H), 6.59–6.39 (m, 2H), 5.32–5.15 (m, 1H), 4.80–4.69 (m, 1H), 4.67–4.54 (m, 1H), 3.92–3.87 (m, 2H), 3.84–3.75 (m, 6H), 2.04–1.93 (m, 1H), 1.39–1.04 (m, 8H), 0.92–0.79 (m, 3H). ^13^C NMR (100 MHz, CDCl_3_) δ 168.03, 164.42, 160.42, 157.82, 134.02, 132.33, 123.53, 119.31, 113.91, 104.26, 98.29, 60.42, 55.39, 55.31, 42.61, 39.88, 31.14, 30.29, 29.37, 28.29, 22.46, 14.02. HRMS (ESI+) m/z calc. for C_26_H_30_N_2_O_5_ 450.2155, found [M + H]^+^ 451.2229. Rf = 0.42 (EtOAc/n-Hex; 1:1, *v*/*v*).

### 4.5. General Procedure for the Synthesis of Monocyclic Beta Lactam Core II (***32***–***34***)

A solution of an appropriate Schiff base (2 EQ) and diazoketone (1 EQ) in 1,2 dimethoxyethane (3 mL) was stirred for 20–30 min at 180 °C in a microwave reactor. The volatiles were then removed in vacuo, and the crude product thus obtained was purified by silica gel column chromatography using EtOAc: Hexane (1:1) as an eluent.

Benzyl ((1-benzyl-2-oxo-4-phenylazetidin-3-yl)methyl)carbamate (**32**), yield: 31%, brown oil. The reaction was carried out according to General Procedure II using *N*-benzyl-1-phenylmethanimine (**9**) (250 mg, 1.28 mmol, 2 EQ) and benzyl (3-diazo-2-oxopropyl)carbamate (**31**) (179 mg, 0.64 mmol, 1 EQ). Product was purified by silica gel column chromatography using EtOAc: n-Hex = 1:1 (*v*/*v*) as eluent. ^1^H NMR (400 MHz, CDCl_3_) δ 7.42 (d, *J* = 1.6 Hz, 1H), 7.40–7.30 (m, 10H), 7.25–7.21 (m, 3H), 7.12 (dd, *J* = 6.9, 2.5 Hz, 2H), 5.18–5.13 (m, 1H), 5.11 (d, *J* = 12.3 Hz, 1H), 4.98 (d, *J* = 12.3 Hz, 1H), 4.83 (d, *J* = 15.0 Hz, 1H), 4.28 (d, *J* = 2.0 Hz, 1H), 3.76 (d, *J* = 14.7 Hz, 1H), 3.64–3.59 (m, 1H), 3.20–3.15 (m, 1H). ^13^C NMR (100 MHz, CDCl_3_) δ 166.69, 156.61, 137.00, 135.31, 134.51, 128.99, 128.79, 128.54, 128.39, 128.33, 128.17, 128.01, 127.73, 126.51, 69.10, 60.36, 57.72, 48.85, 44.57. HRMS (ESI+) m/z calc. for C_25_H_24_N_2_O_3_ 400.1787, found [M + H]^+^ 401.1856. Rf = 0.27. (EtOAc/n-Hex; 1:1, *v*/*v*).

Benzyl ((2-(benzo[b]thiophen-2-yl)-1-benzyl-4-oxoazetidin-3-yl)methyl)carbamate (**33**), yield: 35%, yellow oil. The reaction was carried out according to General Procedure II using 1-(benzo[*b*]thiophen-2-yl)-*N*-benzylmethanimine (**12**) (250 mg, 1.00 mmol, 2 EQ) and benzyl (3-diazo-2-oxopropyl)carbamate (**31**) (117 mg, 0.50 mmol, 1 EQ). Product was purified by silica gel column chromatography using EtOAc: n-Hex = 1:2 (*v*/*v*) as eluent. ^1^H NMR (400 MHz, CDCl_3_) δ 7.81 (d, *J* = 8.4 Hz, 1H), 7.70 (d, *J* = 8.6 Hz, 1H), 7.38–7.30 (m, 13H), 5.13 (s, 2H), 4.98 (d, *J* = 12.2 Hz, 1H), 4.87 (d, *J* = 15.1 Hz, 1H), 4.67 (d, *J* = 1.0 Hz, 1H), 4.33 (s, 1H), 3.86 (d, *J* = 15.1 Hz, 1H). ^13^C NMR (100 MHz, CDCl_3_) δ 167.35, 156.67, 141.88, 139.57, 139.44, 136.25, 135.16, 128.88,128.80, 128.78, 128.75, 128.56, 128.38, 128.21, 128.14, 128.02, 127.87, 124.82, 124.65, 123.68, 123.26, 122.60, 66.99, 60.92, 54.14, 52.36, 44.81. HRMS (ESI+) m/z calc. for C_27_H_24_N_2_O_3_S 456.1508, found [M + H]^+^ 457.1579. Rf = 0.24 (EtOAc/n-Hex; 1:2, *v*/*v*).

Benzyl ((2-(benzo[d][1,3]dioxol-5-yl)-1-benzyl-4-oxoazetidin-3-yl)methyl)carbamate (**34**), yield: 29%, colorless amorphous solid. The reaction was carried out according to General Procedure II using 1-(benzo[d][1,3]dioxol-5-yl)-*N*-benzylmethanimine (**13**) (250 mg, 1.05 mmol, 2 EQ) and benzyl (3-diazo-2-oxopropyl)carbamate (**31**) (123 mg, 0.53 mmol, 1 EQ) Product was purified by silica gel column chromatography using EtOAc: n-Hex = 1:1 (*v*/*v*) as eluent. ^1^H NMR (400 MHz, DMSO-*d*_6_) δ 7.51 (t, *J* = 6.0 Hz, 1H), 7.38–7.25 (m, 9H), 7.16 (d, *J* = 6.7 Hz, 2H), 6.85 (d, *J* = 7.9 Hz, 1H), 6.79 (s, 1H), 6.67 (d, *J* = 7.9 Hz, 1H), 6.01 (d, *J* = 1.3 Hz, 2H), 5.02 (d, *J* = 12.6 Hz, 1H), 4.96 (d, *J* = 12.6 Hz, 1H), 4.65 (d, *J* = 15.6 Hz, 1H), 4.31 (d, *J* = 1.8 Hz, 1H), 3.81 (d, *J* = 15.6 Hz, 1H), 3.70–3.52 (m, 1H), 3.40–3.34 (m, 1H), 3.09 (ddd, *J* = 7.3, 5.1, 1.9 Hz, 1H). ^13^C NMR (100 MHz, DMSO-*d*_6_) δ 167.59, 156.79, 148.26, 147.60, 137.54, 136.50, 132.07, 129.07, 128.79, 128.23, 128.07, 127.81, 120.55, 108.85, 106.72, 101.59, 65.81, 60.66, 58.45, 51.93, 44.12. HRMS (ESI+) m/z calc. for C_26_H_24_N_2_O_5_ 444.1685, found [M + H]^+^ 445.1752. Rf = 0.31 (EtOAc/n-Hex; 1:1, *v*/*v*).

### 4.6. General Procedure for the Synthesis of Monocyclic Beta Lactam Core III (***35***–***37***)

To a solution of an appropriate Schiff base (1 EQ) in dry dichloromethane (2 mL/mmol) was added dry tetrahydrofuran (5 mL/mmol), and the reaction mixture was cooled to −60 °C before mixed acid anhydride (1.3 EQ) was added slowly. Next, triethylamine (1.5 EQ) was added dropwise over a period of 30 min. Upon complete addition, the reaction mixture was allowed to warm to room temperature with stirring overnight. The volatiles were removed in vacuo, and the solid residue thus obtained dissolved in ethyl acetate. The organic phase was washed with 0.1 M HCl (aq), saturated NaHCO_3_ (aq) and brine, dried (Na_2_SO_4_), filtered, then concentrated in vacuo. The crude product thus obtained was then purified by silica gel column chromatography using EtOAc: Hex as eluent or recrystallized from methyl tert-butyl ether.

*tert*-butyl (2-(4-cyanophenyl)-1-(2,4-dimethoxybenzyl)-4-oxoazetidin-3-yl)carbamate (**35**), yield: 11%, colorless amorphous solid. The reaction was carried out according to General Procedure III using 4-(((2,4-dimethoxybenzyl)imino)methyl)benzonitrile (**3**), (14.16 g, 50.5 mmol, 1 EQ), *N*-(*tert*-butoxycarbonyl)glycine (11.5 g, 65.6 mmol, 1.3 EQ) and triethylamine (10.5 mL, 75.7 mmol, 1.5 EQ). Product was purified by silica gel column chromatography using EtOAc: n-Hex = 1:2 (*v*/*v*) as eluent. ^1^H NMR (400 MHz, DMSO-*d6*) δ 7.74 (d, *J* = 8.3 Hz, 2H), 7.52 (d, *J* = 8.4 Hz, 1H), 7.33 (d, *J* = 8.2 Hz, 2H), 7.04 (d, *J* = 8.0 Hz, 1H), 6.46–6.39 (m, 2H), 4.93 (dd, *J* = 8.4, 5.0 Hz, 1H), 4.70 (d, *J* = 5.0 Hz, 1H), 4.43 (d, *J* = 14.5 Hz, 1H), 4.09–3.97 (m, 1H), 3.72 (s, 3H), 3.56 (s, 3H), 1.14 (s, 9H). ^13^C NMR (100 MHz, DMSO-*d*_6_) δ 166.08, 160.96, 158.59, 155.06, 141.81, 132.00, 131.42, 129.23, 119.33, 115.50, 110.46, 105.06, 98.63, 78.80, 62.76, 61.72, 60.22, 55.68, 55.60, 28.24. HRMS (ESI+) m/z calc. for C_24_H_27_N_3_O_5_ 437.1951, found [M + H]^+^ 438.2015. Rf = 0.54 (EtOAc/n-Hex; 1:1, *v*/*v*).

Benzyl (1-(2,4-dimethoxybenzyl)-2-(4-(dimethylamino)phenyl)-4-oxoazetidin-3-yl)carbamate (**36**), yield: 31%, colorless amorphous solid. The reaction was carried out according to General Procedure III using 4-(((2,4-dimethoxybenzyl)imino)methyl)-*N,N*-dimethylaniline (**8**), (1.27 g, 4.25 mmol, 1 EQ), ((benzyloxy)carbonyl)glycine (1. 15 g, 5.52 mmol, 1.3 EQ,) and triethylamine (0.88 mL, 6.37 mmol, 1.5 EQ). Product was purified by silica gel column chromatography using EtOAc: n-Hex = 2:3 (*v*/*v*) as eluent. ^1^H NMR (400 MHz, CDCl_3_) δ 7.39–7.28 (m, 4H), 7.24–6.93 (m, 5H), 6.76–6.60 (m, 2H), 6.48–6.36 (m, 2H), 5.71 (d, *J* = 47.4 Hz, 1H), 5.14 (dd, *J* = 21.9, 9.5 Hz, 1H), 4.94 (d, *J* = 12.0 Hz, 1H), 4.84 (d, *J* = 14.5 Hz, 1H), 4.18 (d, *J* = 20.5 Hz, 2H), 3.79 (app s, 6H), 2.98 (s, 6H). ^13^C NMR (100 MHz, CDCl_3_) δ 167.19, 160.87, 158.60, 153.11, 151.08, 131.78, 128.71, 128.52, 128.42, 128.22, 128.02, 127.72, 116.06, 112.03, 104.09, 98.45, 75.00, 67.19, 67.13, 55.41, 55.25, 48.33, 40.41, 38.28. HRMS (ESI+) m/z calc. for C_28_H_31_N_3_O_5_ 489.2264, found [M + H]^+^ 490.2325. Rf = 0.45 (EtOAc/n-Hex; 1:1, *v*/*v*).

Benzyl (2-(benzo[d][1,3]dioxol-5-yl)-1-(2,4-dimethoxybenzyl)-4-oxoazetidin-3-yl)carbamate (**37**), yield: 33%, colorless amorphous solid. The reaction was carried out according to General Procedure III using 1-(benzo[d][1,3]dioxol-5-yl)-*N*-(2,4-dimethoxybenzyl)methanimine (**14**) (1.85 g, 6.18 mmol, 1 EQ), ((benzyloxy)carbonyl)glycine (1.68 g, 8.04 mmol, 1.3 EQ,) and triethylamine (1.29 mL, 9.27 mmol, 1.5 EQ). Product was precipitated from methyl *tert-*butyl ether. ^1^H NMR (400 MHz, CDCl_3_) δ 7.32–7.25 (m, 4H), 7.15 (d, *J* = 7.2 Hz, 1H), 7.02 (d, *J* = 8.1 Hz, 1H), 6.76 (d, *J* = 8.3 Hz, 1H), 6.62–6.58 (m, 2H), 6.39 (d, *J* = 7.2 Hz, 2H), 5.96 (s, 2H), 5.18 (dd, *J* = 9.4, 5.0 Hz, 1H), 4.98–4.94 (m, 2H), 4.70 (d, *J* = 14.4 Hz, 1H), 4.62 (d, *J* = 4.9 Hz, 1H), 3.99 (d, *J* = 14.4 Hz, 1H), 3.79 (s, 3H), 3.66 (s, 3H). ^13^C NMR (100 MHz, CDCl_3_) δ 166.42, 161.02, 158.56, 155.32, 148.15, 147.66, 135.99, 131.21, 128.46, 128.13, 127.86, 120.64, 115.31, 108.62, 107.02, 104.08, 101.27, 98.40, 77.35, 77.03, 76.72, 66.99, 61.82, 61.13, 55.40, 55.17, 45.81, 39.71, 8.63. HRMS (ESI+) m/z calc. for C_27_H_26_N_2_O_7_ 490.1740, found [M + H]^+^ 491.1803. Rf = 0.28 (EtOAc/n-Hex; 1:1, *v*/*v*).

### 4.7. General Procedure for the Synthesis of Phthalimide Deprotected ß Lactam (***38***–***41***)

#### 4.7.1. Hydrazine Hydrate

3-Amino-4-substituted monocyclic ß-lactams with aromatic substituents:

2-(1-Benzyl-2-oxo-4-(4-(trifluoromethyl)phenyl)azetidin-3-yl)isoindoline-1,3-dione (**19**) (225 mg, 0.44 mmol, 1 EQ) was dissolved in dried methanol and placed under argon atmosphere. Hydrazine hydrate (0.046 mL, 0.76 mmol, 1.7 EQ) was added dropwise. The mixture was stirred for 2 h at room temperature. Then, the solvent was evaporated. Anhydrous methanol and 3 drops of concentrated aqueous HCl were added to the solid. After the solid was completely dissolved again, the solvent was evaporated. The solid was again dissolved in anhydrous methanol and stirred for 16 h at room temperature. The precipitate formed was filtered off and the solvent evaporated. The solid was dissolved in dichloromethane and washed with saturated aqueous NaHCO_3_. The aqueous phase was extracted three times with dichloromethane, the combined organic phases were dried over Na_2_SO_4_ and the solvents evaporated. The deprotected amine was used directly in the next step without purification.

2-(2-((1-(2,4-Dimethoxybenzyl)-2-(4-nitrophenyl)-4-oxoazetidin-3-yl)carbamoyl)benzoyl)hydrazin-1-ide (**38**), colorless amorphous solid. ^1^H NMR (400 MHz, DMSO-*d*_6_) δ 9.37 (s, 1H), 9.04 (d, *J* = 7.9 Hz, 1H), 8.15 (d, *J* = 8.8 Hz, 2H), 7.45 (d, *J* = 8.7 Hz, 2H), 7.39–7.27 (m, 2H), 7.20 (td, *J* = 7.5, 1.4 Hz, 1H), 7.09 (d, *J* = 8.0 Hz, 1H), 6.49–6.39 (m, 4H), 5.39 (dd, *J* = 7.8, 5.1 Hz, 1H), 4.91 (d, *J* = 5.0 Hz, 1H), 4.50 (d, *J* = 14.5 Hz, 1H), 4.30 (s, 1H), 4.17 (d, *J* = 14.4 Hz, 1H), 3.72 (s, 3H), 3.59 (s, 3H). MS (ESI^+^, *m*/*z*) 518.1 ([M − H]^−^). Rf = 0.05 (EtOAc).

1-Benzyl-2-oxo-4-(4-(trifluoromethyl)phenyl)azetidin-3-aminium chloride (**39**), yield: 91%, colorless amorphous solid. ^1^H NMR (400 MHz, DMSO-*d*_6_) δ 8.69 (s, 3H), 7.74 (d, *J* = 8.2 Hz, 2H), 7.59 (d, *J* = 8.2 Hz, 2H), 7.38–7.17 (m, 4H), 5.06 (d, *J* = 5.4 Hz, 1H), 4.92 (d, *J* = 5.4 Hz, 1H), 4.69 (d, *J* = 15.4 Hz, 1H), 4.17 (d, *J* = 15.4 Hz, 1H).^13^C NMR (100 MHz, DMSO-*d*_6_) δ 163.03, 136.83, 135.32, 130.06, 129.63, 129.11, 128.75, 128.19, 125.84, 123.23, 58.90, 57.79, 45.10.HRMS (ESI+) m/z calc. for C_17_H_15_F_3_N_2_O 320.1136, found [M + H]^+^ 321.1208. Rf = 0.61 (EtOAc).

1-(2,4-Dimethoxybenzyl)-2-(4-nitrophenyl)-4-oxoazetidin-3-aminium chloride (**40**), yield: 94%, light brown oil. The reaction was carried out according to the General Procedure using 2-(1-(2,4-dimethoxybenzyl)-2-(4-nitrophenyl)-4-oxoazetidin-3-yl)isoindoline-1,3-dione (**24**) (1.01 g, 2.1 mmol, 1 EQ) and hydrazine hydrate (0.213 mL, 3.5 mmol, 1.7 EQ). Product was used directly in the next step without purification. ^1^H NMR (400 MHz, DMSO-*d*_6_) δ 8.23–8.13 (m, 2H), 7.48–7.38 (m, 2H), 7.03 (dd, *J* = 11.5, 6.3 Hz, 1H), 6.48–6.37 (m, 2H), 4.68 (d, *J* = 5.1 Hz, 1H), 4.45 (d, *J* = 14.7 Hz, 1H), 4.43 (d, *J* = 5.3 Hz, 1H), 4.00 (d, *J* = 14.5 Hz, 1H), 3.71 (s, 3H), 3.56 (s, 3H). ^13^C NMR (100 MHz, DMSO-*d*_6_) δ 170.45, 160.89, 158.58, 147.23, 145.34, 131.29, 129.27, 123.55, 115.89, 105.07, 98.64, 71.70, 66.04, 62.52, 55.66, 55.61. HRMS (ESI+) m/z calc. for C_18_H_19_N_3_O_5_ 357.1325, found [M + H]^+^ 358.1390. Rf = 0.51 (DCM/ iPrOH; 11:1, *v*/*v*).

3-Amino-4-substituted monocyclic ß-lactams with aliphatic substituents:

2-(1-(2,4-Dimethoxybenzyl)-2-isobutyl-4-oxoazetidin-3-yl)isoindoline-1,3-dione (**29**) (200 mg, 0.47 mmol, 1 EQ) in was dissolved in dried methanol and placed under argon atmosphere. Hydrazine hydrate (0.088 mL, 1.42 mmol, 3 EQ) was added dropwise. The mixture was stirred for 2 h at room temperature. Then the solvent was evaporated, and the residue was redissolved in ethyl acetate. The organic phase was washed with saturated aqueous NaHCO_3_ and brine, dried over Na_2_SO_4_ and the solvent was evaporated. Mixture of isomers cis and trans 1-(2,4-dimethoxybenzyl)-2-isobutyl-4-oxoazetidin-3-aminium chloride (**41**), quantitative yield, colorless amorphous solid. ^1^H NMR (400 MHz, CDCl_3_) δ 7.34 (d, *J* = 14.6 Hz, 1H), 6.88 (d, *J* = 8.3 Hz, 1H), 6.80 (d, *J* = 8.4 Hz, 1H), 6.48–6.37 (m, 4H), 6.32 (d, *J* = 13.9 Hz, 1H), 5.09 (dd, *J* = 13.9, 7.3 Hz, 1H), 4.93 (dd, *J* = 14.6, 7.2 Hz, 1H), 4.78 (s, 2H), 4.55 (s, 2H), 3.83 (d, *J* = 6.3 Hz, 6H), 3.79 (d, *J* = 5.4 Hz, 6H), 3.64 (s, 2H), 3.43 (s, 2H), 2.34 (td, *J* = 13.7, 7.0 Hz, 1H), 2.26 (td, *J* = 13.5, 6.8 Hz, 1H), 1.02–0.95 (m, 6H), 0.95–0.89 (m, 6H). ^13^C NMR (100 MHz, CDCl_3_) δ 172.10, 171.86, 160.14, 159.82, 157.74, 157.36, 128.01, 126.41, 124.38, 123.70, 123.44, 119.97, 117.29, 116.10, 104.07, 104.01, 98.37, 98.22, 55.39, 55.34, 55.29, 55.26, 44.05, 43.74, 42.80, 42.13, 29.50, 29.45, 23.11, 22.92. MS (ESI+) m/z calc. for C_16_H_24_N_2_O_3_ 292.1787, found [M + H]^+^ 293.1853. Rf = 0.07 (EtOAc).

#### 4.7.2. Methylhydrazine

2-(1-(2,4-Dimethoxybenzyl)-2-(4-nitrophenyl)-4-oxoazetidin-3-yl) isoindoline-1,3-dione (**24**) (250 mg, 0.51 mmol, 1 EQ) was dissolved in dry methanol and methylhydrazine (0.081 mL, 1.54 mmol, 3 EQ) was added. The reaction was stirred at room temperature. After 4 h, additional methylhydrazine (0.11 mL, 2.12 mmol, 4 EQ) was added and stirred overnight. As the reaction was not yet complete, further methylhydrazine (0.11 mL, 2.12 mmol, 4 EQ) was added, and the reaction was left at room temperature for an additional 72 h. The reaction was allowed to proceed to completion. The organic phase was washed with saturated NaHCO_3_ solution and brine and dried over Na_2_SO_4_. The solvent was evaporated, and the product was purified by silica gel column chromatography using DCM:iPrOH = 11:1 as eluent.

#### 4.7.3. Ethanolamine

2-(1-(2,4-Dimethoxybenzyl)-2-(4-nitrophenyl)-4-oxoazetidin-3-yl) isoindoline-1,3-dione (**24**) (250 mg, 0.51 mmol, 1 EQ) was dissolved in ethyl acetate. Ethanolamine (0.46 mL, 7.7 mmol, 15 EQ) was added, and the reaction mixture was refluxed (80 °C) for 2 h. Then the reaction mixture was cooled to room temperature, and a saturated solution of NaHCO_3_ and additional ethyl acetate were added. The organic phase was washed with brine and dried over Na_2_SO_4_. The solvent was evaporated, and the product was purified by column chromatography using DCM:iPrOH = 11:1 as eluent.

#### 4.7.4. Ethylenediamine

2-(1-(2,4-Dimethoxybenzyl)-2-(4-nitrophenyl)-4-oxoazetidin-3-yl) isoindoline-1,3-dione (24) (250 mg, 0.51 mmol, 1 EQ) was dissolved in ethyl acetate. Ethylendiamine solution (1 M in ethyl acetate; 0.67 mL in 10 mL of ethyl acetate, 10 mmol, 19.5 EQ) was added, and the reaction mixture was stirred overnight at room temperature. After 16 h saturated solution of NaHCO3 and additional ethyl acetate were added. The organic phase was washed with brine and dried over Na_2_SO_4_. The solvent was evaporated and the product purified by silica gel column chromatography using DCM:iPrOH = 11:1 as eluent.

### 4.8. General Procedure for the Synthesis of tert-Butyloxycarbonyl Protected ß Lactam (***43***, ***51***–***53***)

In a flame-dried flask, 3-amino ß-lactam (1 EQ) was dissolved in dry dichloromethane. Triethylamine (1.1 EQ), di-*tert*-butyl dicarbonate (1.5 EQ) and 4-(dimethylamino)pyridine (catalytic amount) were added, and the solution was stirred overnight at room temperature. The solvent was evaporated, and the crude product was purified by silica gel column chromatography using EtOAc: Hex as eluent.

*tert*-butyl (1-benzyl-2-isobutyl-4-oxoazetidin-3-yl)carbamate (**43**), yield: 51%, colorless amorphous solid. The reaction was carried out according to General Procedure using 3-amino-1-(benzyl)-4-isobutylazetidin-2-one (160 mg, 0.69 mmol, 1 EQ), triethylamine (0.11 mL, 0.76 mmol, 1.1 EQ), di-*tert*-butyl dicarbonate (226 mg, 1.03 mmol, 1.5 EQ) and 4-(dimethylamino)pyridine (catalytic amount). Product was purified by silica gel column chromatography using EtOAc: n-Hex = 1:2 (*v*/*v*) as eluent. ^1^H NMR (400 MHz, CDCl_3_) δ 7.37–7.08 (m, 5H), 5.60–5.41 (m, 1H), 5.21–4.93 (m, 1H), 4.88–4.65 (m, 2H), 4.17–3.91 (m, 2H), 2.39–2.19 (m, 1H), 1.52–1.38 (m, 9H), 0.98–0.90 (m, 6H). ^13^C NMR (100 MHz, CDCl_3_) δ 167.47, 155.82, 136.71, 128.52, 127.10, 123.16, 79.76, 47.86, 42.96, 29.54, 28.35, 27.91, 23.01, 22.74. HRMS (ESI+) m/z calc. for C_19_H_28_N_2_O_3_ 332.2100, found [M + H]^+^ 333.2166. Rf = 0.63 (EtOAc/n-Hex; 1:1, *v*/*v*). *tert*-butyl (1-benzyl-2-oxo-4-(4-(trifluoromethyl)phenyl)azetidin-3-yl)carbamate (**51**), yield: 47%, colorless amorphous solid. The reaction was carried out according to General Procedure using 1-benzyl-2-oxo-4-(4-(trifluoromethyl)phenyl)azetidin-3-aminium chloride (**39**) (143 mg, 0.4 mmol, 1 EQ), triethylamine (0.061 mL, 0.44 mmol, 1 EQ), di-*tert*-butyl dicarbonate (130 mg, 0.6 mmol, 1.5 EQ) and 4-(dimethylamino)pyridine (catalytic amount). Product was purified by silica gel column chromatography using EtOAc: n-Hex = 1:2 (*v*/*v*) as eluent. ^1^H NMR (400 MHz, DMSO-*d*_6_) δ 7.64 (d, *J* = 8.1 Hz, 2H), 7.55 (d, *J* = 8.3 Hz, 1H), 7.39 (d, *J* = 8.1 Hz, 2H), 7.34–7.25 (m, 3H), 7.22 (d, *J* = 6.6 Hz, 2H), 5.05 (dd, *J* = 8.3, 5.0 Hz, 1H), 4.85 (d, *J* = 4.9 Hz, 1H), 4.66 (d, *J* = 15.4 Hz, 1H), 4.13 (d, *J* = 15.4 Hz, 1H), 1.39 (s, 9H). ^13^C NMR (100 MHz, CDCl_3_) δ 166.14, 154.27, 138.46, 134.44, 128.99, 128.53, 128.20, 127.83, 125.56, 125.54, 80.39, 62.46, 60.92, 45.15, 27.86. HRMS (ESI+) m/z calc. for C_22_H_23_F_3_N_2_O_3_ 420.1661, found [M + Na]^+^ 443.1550. Rf = 0.58 (EtOAc/n-Hex; 1:1, *v*/*v*).

*tert*-butyl (1-(2,4-dimethoxybenzyl)-2-(4-nitrophenyl)-4-oxoazetidin-3-yl)carbamate (**52**), yield: 60%, colorless amorphous solid. The reaction was carried out according to General Procedure using 1-(2,4-dimethoxybenzyl)-2-(4-nitrophenyl)-4-oxoazetidin-3-aminium chloride (**40**) (143 mg, 0.4 mmol, 1 EQ), triethylamine (0.061 mL, 0.44 mmol, 1 EQ), di-*tert*-butyl dicarbonate (130 mg, 0.6 mmol, 1.5 EQ) and 4-(dimethylamino)pyridine (catalytic amount). Product was purified by silica gel column chromatography using EtOAc: n-Hex = 1:2 (*v*/*v*) as eluent. ^1^H NMR (400 MHz, CDCl_3_) δ 8.18 (d, *J* = 8.6 Hz, 2H), 7.32 (d, *J* = 8.7 Hz, 2H), 7.04 (d, *J* = 8.3 Hz, 1H), 6.39 (dd, *J* = 8.3, 2.3 Hz, 1H), 6.33 (d, *J* = 2.3 Hz, 1H), 5.15 (dd, *J* = 8.1, 5.0 Hz, 1H), 4.86 (d, *J* = 8.1 Hz, 1H), 4.77 (d, *J* = 4.9 Hz, 1H), 4.69 (d, *J* = 14.3 Hz, 1H), 4.13 (d, *J* = 14.3 Hz, 1H), 3.78 (s, 3H), 3.57 (s, 3H), 1.19 (s, 9H). ^13^C NMR (100 MHz, CDCl_3_) δ 165.89, 161.27, 158.50, 154.33, 147.63, 143.08, 131.45, 128.10, 123.40, 114.79, 104.28, 98.36, 80.46, 62.34, 61.42, 55.42, 55.03, 40.41, 27.90. HRMS (ESI+) m/z calc. for C_23_H_27_N_3_O_7_ 457.1849, found [M + H]^+^ 458.1917. Rf = 0.57 (EtOAc/n-Hex; 1:1, *v*/*v*).

*tert*-butyl (1-(2,4-dimethoxybenzyl)-2-isobutyl-4-oxoazetidin-3-yl)carbamate (**53**), yield: 54%, colorless amorphous solid. The reaction was carried out according to General Procedure using 3-amino-1-(2,4-dimethoxybenzyl)-4-isobutylazetidin-2-one (**41**) (138 mg, 0.47 mmol, 1 EQ), triethylamine (0.072 mL, 0.52 mmol, 1.1 EQ), di-*tert*-butyl dicarbonate (155 mg, 0.71 mmol, 1.5 EQ) and 4-(dimethylamino)pyridine (catalytic amount). Product was purified by silica gel column chromatography using EtOAc: n-Hex = 1:1 (*v*/*v*) as eluent. ^1^H NMR (400 MHz, CDCl_3_) δ 6.88 (d, *J* = 8.3 Hz, 1H), 6.47–6.22 (m, 3H), 5.52 (br s, 1H), 5.20–4.90 (m, 1H), 4.80–4.50 (m, 2H), 4.17–3.92 (m, 2H), 3.85–3.75 (m, 6H), 2.38–2.17 (m, 1H), 1.49–1.44 (m, 9H), 0.96 (d, *J* = 6.7 Hz, 3H), 0.93 (d, *J* = 6.7 Hz, 3H). ^13^C NMR (100 MHz, CDCl_3_) δ 167.35, 159.93, 157.42, 154.13, 128.19, 123.12, 104.07, 104.01, 98.26, 83.65, 55.26, 43.29, 43.01, 42.77, 42.40, 29.47, 27.94, 22.79. HRMS (ESI+) m/z calc. for C_21_H_32_N_2_O_5_ 392.2311, found [M + H]^+^ 393.2384. Rf = 0.63 (EtOAc/n-Hex; 1:1, *v*/*v*).

### 4.9. General Procedure for the Synthesis of N1-Benzyl Deprotected ß Lactam (***42***–***44***)

#### Birch Reduction

In a flame-dried flask, Na dispersion in mineral oil (25 wt%, TCI, 6 EQ) and 15-crown-5 (6 EQ) were dissolved in dry tetrahydrofuran. The solution was warmed to room temperature under argon and stirred vigorously for 5 min. Then, the reaction mixture was cooled to 0 °C before a solution of ß-lactam (1 EQ), and isopropanol (6 EQ) in tetrahydrofuran was slowly added. After 15 min, the reaction was stopped by the addition of a saturated aqueous solution of NaHCO_3_ and diethyl ether. The aqueous phase was extracted with diethyl ether (2 × 30 mL). The combined organic phases were dried (Na_2_SO_4_), filtered, then concentrated in vacuo. The crude product thus obtained was purified by silica gel column chromatography using EtOAc:Hex as eluent.

*N-(2,4-dimethoxybenzyl)-3-(p-tolyl)propanamide* (**42**), colorless amorphous solid. ^1^H NMR (400 MHz, CDCl_3_) δ 7.10 (d, *J* = 8.0 Hz, 1H), 7.05 (s, 4H), 6.46–6.37 (m, 2H), 5.76 (br s, 1H), 4.32 (d, *J* = 5.7 Hz, 2H), 3.80 (s, 3H), 3.77 (s, 3H), 2.94–2.85 (m, 2H), 2.47–2.38 (m, 2H), 2.30 (s, 3H). ^13^C NMR (100 MHz, CDCl_3_) δ 171.69, 160.48, 158.52, 137.86, 135.54, 130.53, 129.12, 128.21, 118.88, 103.88, 98.55, 55.41, 55.28, 38.86, 38.72, 31.29, 21.00. HRMS (ESI+) m/z calc. for C_19_H_23_NO_3_ 313.1678, found [M + H]^+^ 314.1745. Rf = 0.51 (EtOAc/n-Hex; 1:1, *v*/*v*).

*tert*-butyl (2-isobutyl-4-oxoazetidin-3-yl)carbamate (**44**), yield: 89%, transparent oil. The reaction was carried out according to General Procedure using Na dispersion in mineral oil (25 wt%, TCI, 131 mg, 13.5 mmol, 6 EQ),15-crown-5 were (0.283 mL, 13.5 mmol, 6 EQ), *tert*-butyl (1-benzyl-2-isobutyl-4-oxoazetidin-3-yl) carbamate (**43**) (75 mg, 2.25 mmol, 1 EQ) and isopropanol (0,109 mL, 13.5 mmol, 6 EQ). Product was purified by silica gel column chromatography using EtOAc: n-Hex = 1:1 (*v*/*v*) as eluent.. ^1^H NMR (400 MHz, CDCl_3_) δ 7.64 (br s, 1H), 6.69 (ddd, *J* = 14.3, 10.5, 1.2 Hz, 1H), 5.19 (dd, *J* = 14.3, 7.0 Hz, 1H), 5.15–5.07 (m, 1H), 3.82 (d, *J* = 5.9 Hz, 2H), 2.33 (dqd, *J* = 13.5, 6.8, 1.3 Hz, 1H), 1.47 (s, 9H), 1.01 (s, 3H), 1.00 (s, 3H). HRMS (ESI+) m/z calc. for C_12_H_22_N_2_O_3_ 242.1630, found [M + H]^+^ 243.1685. Rf = 0.36 (EtOAc/n-Hex; 1:1, *v*/*v*).

### 4.10. General Procedure for the Synthesis of N1-Dimethoxybenzyl Deprotected ß Lactam (***46***–***50***)

#### Cerium Ammonium Nitrate

ß-Lactam (1 EQ) was dissolved in acetonitrile (25 mL/mmol) and distilled water (20 mL/mmol) and placed under argon. The solution was cooled to −10 °C with a sodium chloride ice bath. Cerium ammonium nitrate (3 EQ) was dissolved in distilled water and added dropwise to the vigorously stirring reaction mixture. The reaction was stirred at −10 °C for 1–2 h and then transferred to a separation funnel containing diethyl ether and saturated aqueous NaHCO_3_. The aqueous phase was washed with diethyl ether. The combined organic phases were dried over Na_2_SO_4,_ and the solvents were evaporated. The solid was purified by silica gel column chromatography, using the gradient EtOAc: Hex as eluent.

*tert*-butyl (1-(2,4-dimethoxybenzoyl)-2-(4-nitrophenyl)-4-oxoazetidin-3-yl)carbamate (**44**), light orange amorphous solid. ^1^H NMR (400 MHz, CDCl_3_) δ 8.25 (d, *J* = 8.7 Hz, 2H), 7.55–7.51 (m, 1H), 7.52 (d, *J* = 8.4, 2H), 6.61–6.51 (m, 2H), 5.59 (d, *J* = 6.4 Hz, 1H), 5.36–5.27 (m, 1H), 4.70 (d, *J* = 8.4 Hz, 1H), 3.91 (s, 3H), 3.88 (s, 3H), 1.28 (s, 9H). ^13^C NMR (100 MHz, CDCl_3_) δ 164.61, 163.87, 160.10, 154.18, 147.80, 141.44, 132.04, 127.90, 123.80, 115.51, 105.18, 98.65, 81.20, 60.79, 59.56, 55.93, 55.58, 27.95. HRMS (ESI+) m/z calc. for C_23_H_25_N_3_O_8_ 471.1642, found [M + Na]^+^ 494.1532. Rf = 0.43 (EtOAc/n-Hex; 2:1, *v*/*v*).

2-(2-Oxo-4-(4-(trifluoromethyl)phenyl)azetidin-3-yl)isoindoline-1,3-dione (**46**), yield: 65%, colorless amorphous solid. The reaction was carried out according to General Procedure using 4-(1-(2,4-dimethoxybenzyl)-3-(1,3-dioxoisoindolin-2-yl)-4-oxoazetidin-2-yl)benzonitrile (**21**) (156 mg, 0.31 mmol, 1 EQ) and cerium ammonium nitrate (502 mg, 0.92 mmol, 3 EQ). Product was purified by silica gel column chromatography using EtOAc: n-Hex = 1:1 (*v*/*v*) as eluent. ^1^H NMR (400 MHz, CDCl_3_) δ 7.72–7.60 (m, 4H), 7.54–7.41 (m, 4H), 7.28 (br s, 1H), 5.71 (dd, *J* = 5.4, 1.8 Hz, 1H), 5.26 (d, *J* = 5.4 Hz, 1H). ^13^C NMR (100 MHz, CDCl_3_) δ = 166.66, 164.59, 138.82, 134.48, 131.00, 130.45, 130.13, 127.11, 125.43, 123.62, 60.37, 57.23. HRMS (ESI+) m/z calc. for C_18_H_11_F_3_N_2_O_3_ 360.0722, found [M + H]^+^ 361.0791. Rf = 0.43 (EtOAc/n-Hex; 1:1, *v*/*v*).

2-(2-(3-Bromo-4-fluorophenyl)-4-oxoazetidin-3-yl)isoindoline-1,3-dione (**47**), yield: 40%, light yellow amorphous solid. The reaction was carried out according to General Procedure using 2-(2-(4-bromo-3-fluorophenyl)-1-(2,4-dimethoxybenzyl)-4-oxoazetidin-3-yl)isoindoline-1,3-dione (**22**) (151 g, 0,29 mmol, 1 EQ) and cerium ammonium nitrate (473 mg, 0.86 mmol, 3EQ). Product was purified by silica gel column chromatography using EtOAc: n-Hex = 2:1 (*v*/*v*) as eluent. ^1^H NMR (400 MHz, CDCl_3_) δ 7.76–7.65 (m, 4H), 7.53 (dd, *J* = 6.4, 2.0 Hz, 1H), 7.29–7.23 (m, 1H), 7.03 (br s, 1H), 6.99 (t, *J* = 8.4 Hz, 1H), 5.63 (dd, *J* = 5.3, 1.9 Hz, 1H), 5.14 (d, *J* = 5.3 Hz, 1H). ^13^C NMR (100 MHz, CDCl_3_) δ 166.65, 164.37, 159.93, 157.46, 134.52, 132.15, 132.12, 131.98, 131.04, 127.46, 127.38, 123.69, 116.66, 116.44, 109.29, 109.07, 60.45, 56.58. HRMS (ESI+) m/z calc. for C_17_H_10_BrFN_2_O_3_ 387.9859, found [M + H]^+^ 388.9936. Rf = 0.43 (EtOAc/n-Hex; 2:1, *v*/*v*).

*tert*-butyl (2-(4-nitrophenyl)-4-oxoazetidin-3-yl)carbamate (**48**), yield: 51%, light red amorphous solid. The reaction was carried out according to General Procedure using *tert*-butyl (1-(2,4-dimethoxybenzyl)-2-(4-nitrophenyl)-4-oxoazetidin-3-yl)carbamate (**52**) (2.5 g, 5.4 mmol, 1 EQ) and cerium ammonium nitrate (9 g, 16.4 mmol, 3 EQ). Product was purified by silica gel column chromatography using the gradient EtOAc: Hex = 1:1 to 4:1 as eluent. ^1^H NMR (400 MHz, DMSO-*d*_6_) δ 8.71 (br s, 1H), 8.22 (d, *J* = 8.7 Hz, 2H), 7.46 (d, *J* = 8.6 Hz, 2H), 7.42 (d, *J* = 8.4 Hz, 1H), 5. 41–5.36 (m, 1H), 5.05–4.93 (m, 1H), 1.18 (s, *J* = 12.9 Hz, 9H). ^13^C NMR (100 MHz, DMSO-*d*_6_) δ 167.10, 155.14, 147.19, 145.99, 128.87, 123.40, 78.84, 63.68, 56.77, 28.27. HRMS (ESI+) m/z calc. for C_14_H_17_N_3_O_5_ 307.1168, found [M + H]^+^ 308.1240. Rf = 0.23 (EtOAc/n-Hex; 2:1, *v*/*v*).

2-(2-(4-(Methylsulfonyl)phenyl)-4-oxoazetidin-3-yl)isoindoline-1,3-dione (**49**), yield: 43%, yellow amorphous solid. The reaction was carried out according to General Procedure using 2-(1-(2,4-Dimethoxybenzyl)-2-(4-(methylsulfonyl)phenyl)-4-oxoazetidin-3-yl)isoindoline-1,3-dione (**25**) (72 mg, 0.14 mmol, 1 EQ) and cerium ammonium nitrate (228 mg, 0.42 mmol, 3 EQ). Product was purified by silica gel column chromatography using EtOAc: n-Hex = 2:1 (*v*/*v*) as eluent. ^1^H NMR (400 MHz, CDCl_3_) δ 7.81 (d, *J* = 8.4 Hz, 2H), 7.74–7.64 (m, 4H), 7.54 (d, *J* = 8.2 Hz, 2H), 7.14 (br s, 1H), 5.72 (dd, *J* = 5.5, 1.9 Hz, 1H), 5.27 (d, *J* = 5.5 Hz, 1H), 2.90 (s, 3H). ^13^C NMR (100 MHz, CDCl_3_) δ 166.58, 164.18, 141.21, 140.20, 134.62, 130.95, 127.79, 127.56, 123.74, 60.48, 57.18, 44.33. HRMS (ESI+) m/z calc. for C_18_H_14_N_2_O_5_S 370.0623, found [M + Na]^+^ 393.0530. Rf = 0.33 (EtOA).

2-(2-(Furan-2-yl)-4-oxoazetidin-3-yl)isoindoline-1,3-dione (**50**), yield: 15%, light brown oil. The reaction was carried out according to General Procedure 2-(1-(2,4-dimethoxybenzyl)-2-(furan-2-yl)-4-oxoazetidin-3-yl)isoindoline-1,3-dione (**26**) (206 mg, 0.48 mmol, 1 EQ) and cerium ammonium nitrate (0.84 g, 1.43 mmol, 3 EQ). Product was purified by silica gel column chromatography using EtOAc: n-Hex = 2:1 (*v*/*v*) as eluent. ^1^H NMR (400 MHz, DMSO-*d*_6_) δ 8.04–7.80 (m, 4H), 7.02 (dd, *J* = 10.6, 3.7 Hz, 1H), 6.91 (d, *J* = 7.3 Hz, 1H), 6.08 (dd, *J* = 10.6, 1.4 Hz, 1H), 5.87 (d, *J* = 6.0 Hz, 1H), 5.64 (dd, *J* = 6.0, 3.7 Hz, 1H), 4.55 (d, *J* = 6.0 Hz, 1H). ^13^C NMR (100 MHz, DMSO-*d*_6_) δ 191.40, 167.88, 165.11, 146.54, 135.84, 131.53, 128.34, 124.56, 67.63, 56.54, 53.78. HRMS (ESI+) m/z calc. for C_15_H_10_N_2_O_4_ 282.0641, found [M + H]^+^ 283.0721. Rf = 0.33 (EtOAc/n-Hex; 2:1, *v*/*v*).

### 4.11. General Procedure for the Synthesis of tert-Butyloxycarbonyl Deprotected ß Lactam (***54***)

#### With Use of Trifluoroacetic Acid

*tert*-butyl (2-(4-nitrophenyl)-4-oxoazetidin-3-yl)carbamate (**48**) (150 mg, 0.5 mmol, 1 EQ) was dissolved in dry dichloromethane (2 mL), anisole (0.49 mL, 4.5 mmol, 9 EQ) was added, and the solution was cooled to −5 °C on a sodium chloride ice bath. Trifluoroacetic acid (1.53 mL, 20 mmol, 40 EQ) was added, and the solution was slowly warmed to room temperature. After stirring for 1.5 h, the solvent and the excess trifluoroacetic acid were evaporated. The residue was precipitated from methyl *tert*-butyl ether. The solid was used in the next step without further purification.

2-(4-Nitrophenyl)-4-oxoazetidin-3-aminium trifluoroacetate (**54**), yield: 83%, brown oil. ^1^H NMR (400 MHz, DMSO-*d*_6_) δ 9.24 (s, 1H), 8.46 (br s, 3H), 8.30 (d, *J* = 8.8 Hz, 2H), 7.67 (d, *J* = 8.6 Hz, 2H), 5.19 (d, *J* = 5.4 Hz, 1H), 4.86 (dd, *J* = 5.4, 1.8 Hz, 1H). ^13^C NMR (100 MHz, DMSO-*d*_6_) δ 163.43, 148.06, 142.37, 129.63, 123.92, 59.90, 49.18. HRMS (ESI+) m/z calc. for C_9_H_9_N_3_O_3_ 207.0644, found [M + H]^+^ 208.0716. Rf = 0.08 (EtOAc/n-Hex; 9:1, *v*/*v*).

## Figures and Tables

**Table 1:** Unsuccessful and successful methods for removal of N-benzyl protecting group and *N*-dimethoxybenzyl protecting group.

N-1 Deprotection Method	Benzyl Protecting Group	Dimethoxybenzyl Protecting Group
Hydrogenation using Pd/C	No product detected (Starting material isolated)	/
Hydrogenation using cyclohexene/Pd (OH)_2_	No product detected (Starting material isolated)	/
High pressure using hydrogenation Pd/C at 30 bar	Traces	/
Ammonia-free Birch reduction	*C-4 Aromatic*: No product; *C-4 Aliphatic*: High yields 89%	No product detected (Side product detected: **42**)
Acid hydrolysis (CF_3_COOH)	/	No product detected
Acid hydrolysis (TsOH)	/	No product detected
Cerium ammonium nitrate ((NH_4_)_2_Ce(NO_3_)_6_)	/	15%–65% (Side product detected: **45**)
Potassium persulfate (K_2_S_2_O_8_)	/	Up to 10%
Ammonium Persulfate ((NH_4_)_2_S_2_O_8_)	/	Up to 13%

## Data Availability

Not applicable.
